# Drinking Water Utility-Level Understanding of Climate Change Effects to System Reliability

**DOI:** 10.1021/acsestwater.3c00091

**Published:** 2023-07-13

**Authors:** Zia J. Lyle, Jeanne M. VanBriesen, Constantine Samaras

**Affiliations:** † Department of Civil and Environmental Engineering, Carnegie Mellon University, 5000 Forbes Avenue, Pittsburgh, Pennsylvania 15213, United States; ‡ Department of Engineering and Public Policy, Carnegie Mellon University, 5000 Forbes Avenue, Pittsburgh, Pennsylvania 15213, United States

**Keywords:** Drinking water, Climate change, Reliability, Utility management, Adaptation, Resilience

## Abstract

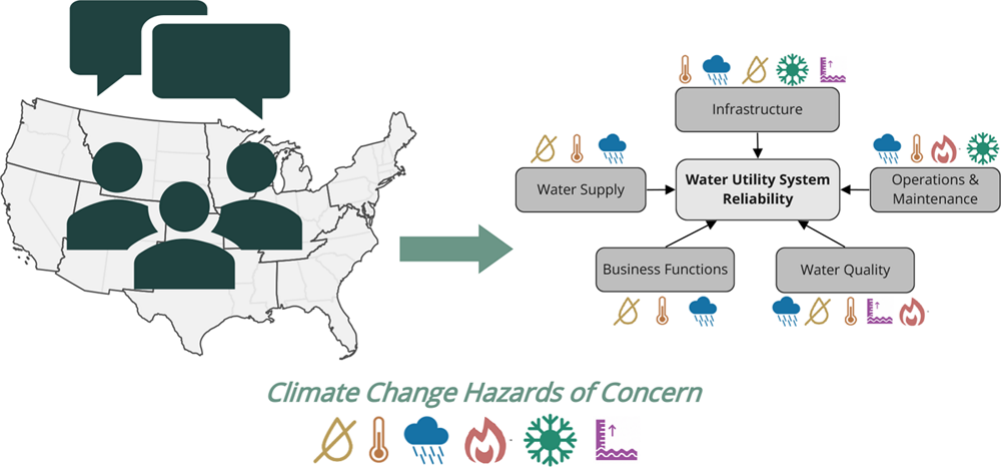

Climate change hazards, including increased temperatures, drought, sea level rise, extreme precipitation, wildfires, and changes in freeze–thaw cycles, are expected to degrade drinking water utility system infrastructure and decrease the reliability of water provision. To assess how drinking water utility manager perceptions of these risks affect utility planning, 60 semistructured interviews were conducted with utilities of various sizes, source water supplies, and United States geographical regions. This study analyzes these interviews (1) to evaluate which climate hazards are of primary concern to drinking water managers, (2) to develop a mental model framework for assessing utility-level understanding of climate change risks to system reliability, and (3) to examine the status of current water utility adaptation planning. The results show that concern and awareness of climate hazard risks vary geographically and are grounded in historical exposure; some participants do not believe climate change will influence their system’s overall reliability. When considering climate change risks, utility managers tend to focus on effects to water supply and infrastructure, as opposed to changes in operations and maintenance, water quality, or business functions. Most surveyed utilities do not have comprehensive climate adaptation plans despite federal and professional recommendations. The range of beliefs and actions concerning climate adaptation planning indicates that utilities need directed guidance, and policymakers should consider including climate hazards and projections as part of required utility risk and resilience assessments.

## Abstract 2

Interviews with drinking water utility managers reveal understanding of climate hazards and status of climate adaptation planning.

## 1 Introduction

Climate change poses significant challenges for drinking water utility infrastructure and business operations. It disrupts the traditional water management expectation that historical variability of natural systems will remain consistent into the future.^1^ Instead, climate change will result in increased temperatures, sea level rise, extreme precipitation, wildfires, and changes in freeze–thaw cycles, which may degrade drinking water system infrastructure.^2−5^ Assessments of the magnitude of climate risk and the effectiveness of utility planning require evaluations of how drinking water utility managers consider the effects of climate-induced changes on system operations.

Water utilities are considered highly reliable organizations, as they have outstanding safety records^6^ and meet consumer demands for water quantity and quality under normal and emergency conditions.^7^ Although drinking water utilities are considered reliable, there is a lack of consistency in reliability of service standards.^8^ Regulations require tracking water quality compliance violations but not level of service metrics, like length or frequency of outages. There are a range of indicators used to benchmark water utilities, including service outages, maintenance needs, and customer satisfaction indices, but most are self-reported to professional organizations^9^ and few are regulated.^10^ Water utility reliability requires management of the engineering, operations, and maintenance of infrastructure systems, in addition to business functions including finance, public and government affairs, and water supply planning and forecasting.^4^ Planning timelines are long, and government legislation and associated regulations are significant drivers for utility operations. Adapting water utilities for climate change to maintain reliability requires considering gradual changes in weather patterns as well as increases in the frequency and magnitude of extreme events.^11^ Gradual changes can affect maintenance and component reliability, while extreme events require updates to emergency response plans and long-term changes in designs and operations.

Effects of climate change on drinking water quality and quantity have been documented for decades.^12^ Drought and hotter temperatures reduce available water resources; increased evapotranspiration rates in natural and engineered water bodies and reduced snowpack decrease multiple forms of water storage.^5^ Increased temperatures additionally shorten lifetimes of water utility equipment and infrastructure components.^3,13^ Sea level rise may cause saltwater intrusion into groundwater aquifers, affecting water quality and degrading buried infrastructure.^14,15^ Changes in the intensity and frequency of rainfall events may overwhelm treatment capacity, further degrade water quality, and flood utility assets.^3^ Wildfires can also deteriorate water quality and damage infrastructure.^16−18^ See Supporting Information (SI) Section 1 for more details about the potential effects of climate hazards on water utilities. These effects are expected to alter system operations, and thus utility planning is required to ensure systems maintain necessary levels of reliability.

While these potential effects of climate change on water utility system reliability have been well documented,^19−21^ it is unclear whether utility managers are aware of the scope of the climate risk facing their systems. Although guidance and tools for climate resilience exist,^3,4,22−24^ literature on how utilities consider climate risk is limited to case studies. Water utilities that are considered leaders in the climate adaptation space have utilized scenario planning, constructed new water supplies, and developed emergency response plans.^25,26^ Prior case studies indicate water utility climate planning efforts focus on mitigating hydrologic changes to water resources,^27^ rather than a recommended focus on effects to business functions and operations.^4^ Planning within the water sector has focused on extreme events; the more gradual, slow-moving climate hazards are lower priorities.^21^ Overall utility resilience efforts have focused on mitigating drainage and power issues and addressing interdependencies between water and electricity sectors; utilities use general hazard mitigation funding to invest in pumps, generators, and drainage projects.^28^ Determining the perceptions of water utility managers relative to climate risk and system reliability is needed to inform policy decisions and utility planning activities.

Behavioral evidence can provide these insights into gaps between fully informed decision-making recommendations and current considerations of climate risk.^29^ Given that infrastructure managers must develop procedures to adapt to climate change under uncertainty,^30^ qualitative methods, like semistructured interviews with utility managers, can reveal the nature of current risk perceptions and develop practical, generalizable recommendations.^31−34^ Behavioral research characterizes individual perceptions, as well as professional and value judgments about which climate hazards are considered risks for utilities.^35^ This reveals gaps between prescriptive recommendations for climate planning (i.e., what should be done) and descriptive realities (i.e., what is currently being done).^29^ Mental models, or internal representations of one’s surroundings, specifically structure belief systems and decision-making around complex topics like climate adaptation.^36,37^ Expert mental models aggregate individual mental models to generalize about that population.^37^ This approach has been used previously to characterize climate risk and adaptation perceptions^38−40^ and utility emergency response actions.^41^

The objective of this work is to explore how utility-manager-level perceptions of climate risk influence adaptation planning and decision-making. Semistructured interviews with managers at medium and large drinking water utilities are used to (1) identify climate hazards of concern and whether these hazards affect facets of system reliability, (2) build a utility manager mental model of climate-induced changes to system reliability, and (3) catalog climate adaptation planning efforts.

## 2 Methods

This study uses semistructured interviews to develop a descriptive mental model of how drinking water utility decision-makers evaluate potential climate-induced changes to system reliability (see SI Figure S2). Semistructured interviews were chosen for this study because they an appropriate method of collecting qualitative data when studying people’s perceptions about complex, novel issues and when there are theoretical gaps in the literature.^42^ This approach has been used previously to characterize water utility perspectives.^32,33,43^

### 2.1 Data Collection

Semistructured interviewers were conducted with 60 water utility managers/directors across the continental United States (US) under approval of the Carnegie Mellon University institutional review board. As the drinking water sector lacks an operational or regulatory definition of system reliability,^8^ the first portion of the interview focused on developing the participant’s framing of the term. Participants were asked how they define reliability and which components of reliability (i.e., system outages, water quality issues, maintenance needs, and customer satisfaction) they thought were important. The majority of the interview then explored which natural hazards are of top concern for reliability and how risks to reliability are perceived to be changing; this was meant to elicit information about how utilities are evaluating and responding to hazards exacerbated by climate change without explicitly mentioning the term. Waiting to use the term “climate change” until the final questions was designed to reduce the influence of participant biases and heuristics associated with political framings and misunderstandings of climate change.^44,45^ In the final section, participants were asked how climate change specifically could affect system reliability and whether their utility had a climate adaptation plan. Participants were asked to use framings of reliability from earlier in the interview and were not prompted with specific lists of climate hazards or utility functions. Interview questions were tested for clarity through a survey distributed at the April 2022 Pennsylvania American Water Works Association (AWWA) conference in Harrisburg, PA and informed by work concerning electric utility resilience.^46^ See SI Section 2 for interview protocol. Interviews were conducted virtually over Zoom from June 2022 to August 2022 and lasted approximately 1 h. Each participant was interviewed individually by the same interviewer.

**Figure 1:**
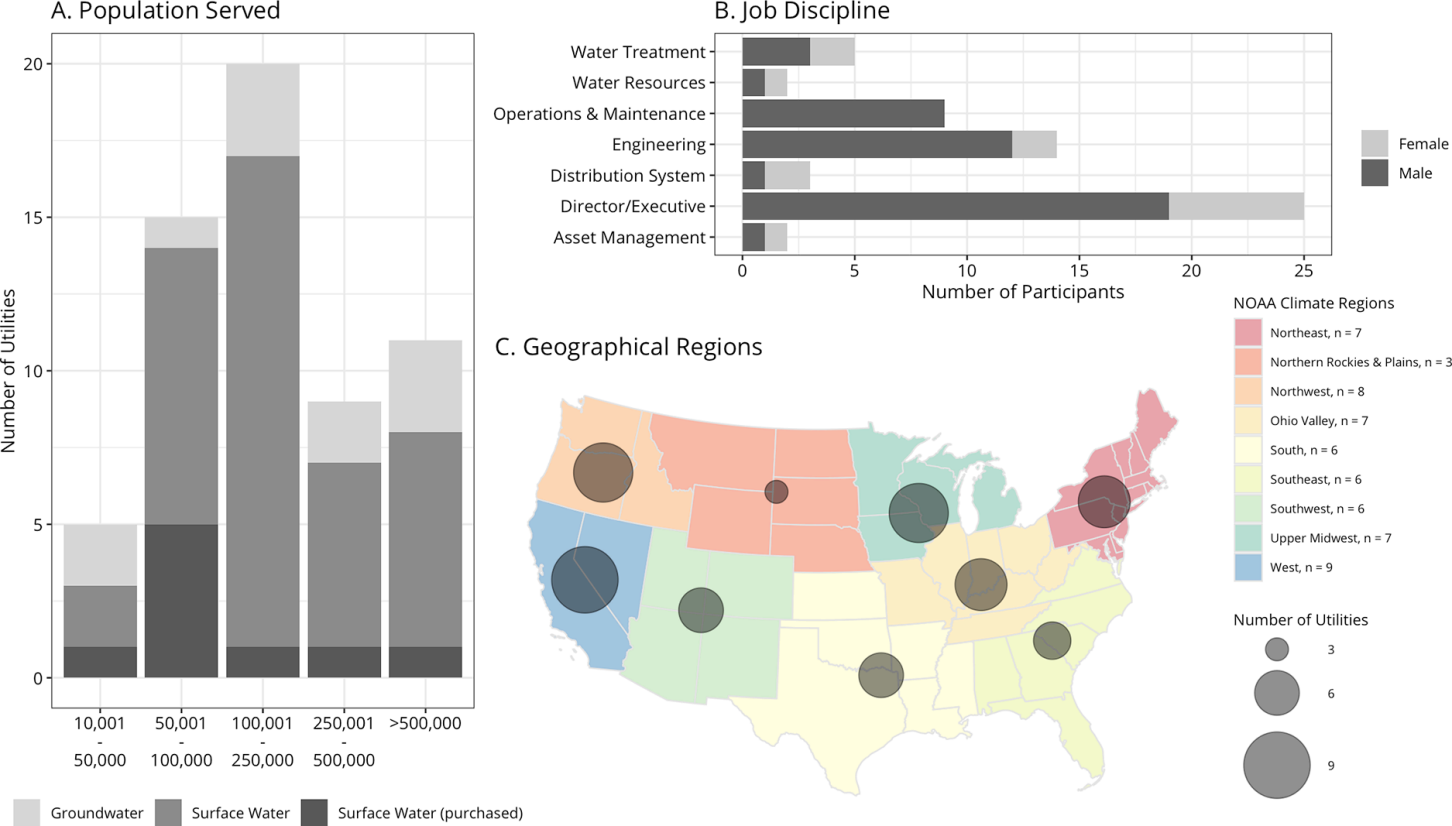
Characteristics of the interview participants.

A convenience sampling technique was used to target participants meeting certain criteria.^47,48^ The targeted sample population was medium and large community water systems (>10,000 customers) to ensure planning and engineering design is completed within the utility organization (rather than by external consultants as is typical at smaller utilities) and that each system has similar minimum infrastructure components (i.e., water treatment plant). Given this focus, most interviewed utility managers worked at suburban or urban surface water systems, as those tend to serve larger populations. Given regional differences in climate change hazards and adaptation actions,^40,49^ utility managers in different geographic locations are expected to have different hazard concerns. Utilities from nine NOAA climate regions were interviewed, and regional differences in their answers were noted.^50^ Information about the population served, source water type, and NOAA climate region was collected to analyze differences among water utilities (Figure 1). Participants all held manager- or director-level positions within their water utility but represented a range of job disciplines (Figure 1, Panel B). Interviews were conducted until a geographical distribution was achieved; at least six utilities were interviewed in each climate regions (excluding Northern Rockies and Plains due to sampling limitations). The Safe Drinking Water Information System database provided contact information and system properties.

### 2.2 Data Analysis

A hybrid content analysis was used; interviews were deductively coded using existing frameworks, such as identified climate hazards, and additional inductive coding allowed emergent themes to be quantified.^31,33,34,51^ The unit of analysis was the participant’s complete response to an interviewer’s question. Dedoose, a web-based application for mixed-methods analysis, was used to code the interview transcripts.^52^ The deductive coding dictionary was developed using a directed approach for content analysis, which starts with a theory or relevant research findings as guidance for initial codes.^53^ The inductive coding process was iterative; multiple rounds of coding analysis allowed a range of themes to emerge.^54,55^ The coding was nonexclusive; for example, participants could identify multiple potential effects to system reliability while expressing that they were unconcerned about those effects on their own system. One researcher coded all excerpts, and another researcher coded six excerpts (10% of the total) to check intercoder reliability using the Mezzich’s κ statistic, which captures overlap for nonexclusive coding.^56^ The coders have engineering backgrounds and no utility operator field experience so nuances in meanings might have been missed, though adhering to a consistent coding protocol helped reduced coder biases.^51^ The achieved κ value of 0.67 is considered satisfactory.^56,57^ See SI Section 3 for a coding dictionary.

A generalized water utility manager mental model was then developed using the emergent codes specifically concerning climate change effects on utility system reliability. This was an expert model, which captures combined beliefs of technical specialists (water utility managers)^37^ and thus reflects aggregated perspectives of individuals, not entire organizations. Mental models are typically illustrated using influence diagrams showing nodes representing variables and arrows indicating directions of influence.^58^

In addition to developing an aggregated mental model capturing perceptions of utility managers about effects of climate change on system reliability, various characteristics of the water utilities were used in the analysis of participant responses. Climate change exposure and adaptation progress vary geographically, so regional perspectives on hazards of concern were assessed.^40,49^ The utility’s source water, either surface water or groundwater, could affect its exposure to different climate hazards and thus was considered a possible explanatory variable in the analysis.^12,59^ Also, a system’s adaptive capacity, or ability to respond to changes, depends on its surrounding sociotechnical influences^60^ and is directly related to population size and resource availability.^61^ Thus, these characteristics were noted and considered in the analysis.

### 2.3 Limitations

The qualitative protocol and sample of interviewed utilities were chosen to generally determine how drinking water utility managers consider and address climate change risks, but these choices limit the implications of the study results. Interviews with individual utility managers sought to capture how drinking water professionals approach climate risk decision-making. These individual perceptions do not reflect a complete picture of institutional practices within a utility, and this limits broad, organizational-level conclusions about water utilities from this work. While the interviewed utility managers held a range of job disciplines, the largest represented group was general managers or directors who have more business-oriented responsibilities than other roles, potentially overemphasizing long-term concerns over day-to-day issues. Additionally, the semistructured nature of the interview protocol (i.e., participants were not asked explicitly to quantify and compare climate risks for each utility function) limits conclusions about specific vulnerabilities facing water utilities. Finally, the interviewed sample does not represent all drinking water utilities in the US. The sampling criteria to include only utilities serving at least 10,000 customers means surface water systems are over-represented, and there were fewer interviewed participants from the Northern Rockies and Plains region, where there are fewer utilities of this size. This limited sample may skew results toward issues faced by large, urban, and surface water utilities. Nevertheless, the range of discussed climate hazards and effects on system reliability may offer broader insights to all utility professionals.

## 3 Results and Discussion

The 60 interviewed water utility managers/directors represent a geographic distribution of unique drinking water utilities that serve a combined 19 million people.

### 3.1 Framing System Reliability

Most participants defined reliability as continuity of service, emphasizing redundancy within the system and being able to meet level of service goals regardless of emergency conditions. Participants were split in how they regarded the time aspect of reliability: 30% said they cared more about long-term reliability than short-term reliability; 28% said the opposite; and 42% said they cared about both. Participants discussed short-term emergencies as influencing the development of longer-term planning efforts. In terms of utility functions, 48% identified water quality as the most important function to preserving system reliability, given it concerns the health and safety of customers and is the primary regulatory requirement. Twenty-three percent said water supply was the function with the most influence on system reliability, and 22% said operations and maintenance was most influential. Most participants (45 out of 60) believed that their utility is more reliable today than a decade ago, which was attributed to continual investments in infrastructure, regulatory changes, and technology improvements. Nine participants attributed decreases in reliability to climate change: each identified droughts affecting water supplies.

### 3.2 Establishing Links between Natural Hazards and Changes in Reliability

**Figure 2:**
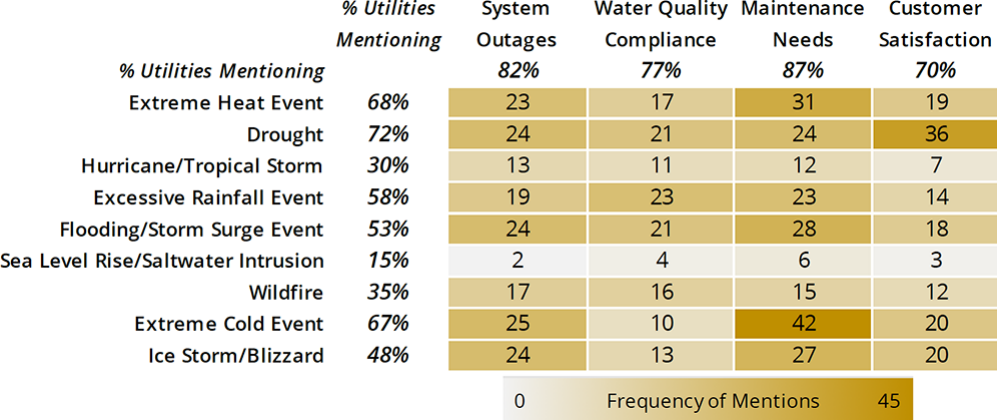
Hazard–reliability matrix for all participants (*n* = 60). Shading indicates the frequency of hazard (vertically along the left) and component of reliability (across the top) pair mentions from all interviewed participants.

Utility managers were asked to identify hazards they expected to pose risks and which component of reliability they expected to be affected; these included system outages, water quality issues, maintenance needs, and customer satisfaction (Figure 2). Climate change was not explicitly linked to any of the hazards at this point of the interviews, although three utilities connected it to the prompt.

Figure 2 shows the aggregated hazard–reliability matrix with values representing the number of utilities mentioning the hazard–reliability pair. Dark shading indicates over half of the participants mentioned that the hazard could affect the component of reliability, while lighter shading represents almost no mentions of the hazard–reliability pair. A majority of utilities expressed potential risks to their system from droughts (72%, 43 of the 60 participants), extreme heat events (68%), extreme cold events (58%), and flooding or storm surge events (53%). Participants were least concerned about sea level rise/saltwater intrusion, as it only affects coastal utilities.

Each reliability component was mentioned by at least 70% of participants as potentially being affected by at least one hazard. Maintenance needs were the most mentioned component that could be affected; this included examples like pipe breaks in the winter, responding to an extreme event, and degradation of equipment and infrastructure. The participants identified connections between some of the reliability components; for instance, that system outages could cause changes in customer satisfaction. Participants also discussed how some hazards might pose risks to water quality but not that they would rise to the level of a compliance violation. An example is taste and odor changes, which could also affect customer satisfaction. Some participants noted that while their system was at risk of various natural hazards, they had resilience measures in place to mitigate the consequences to system reliability.

**Figure 3:**
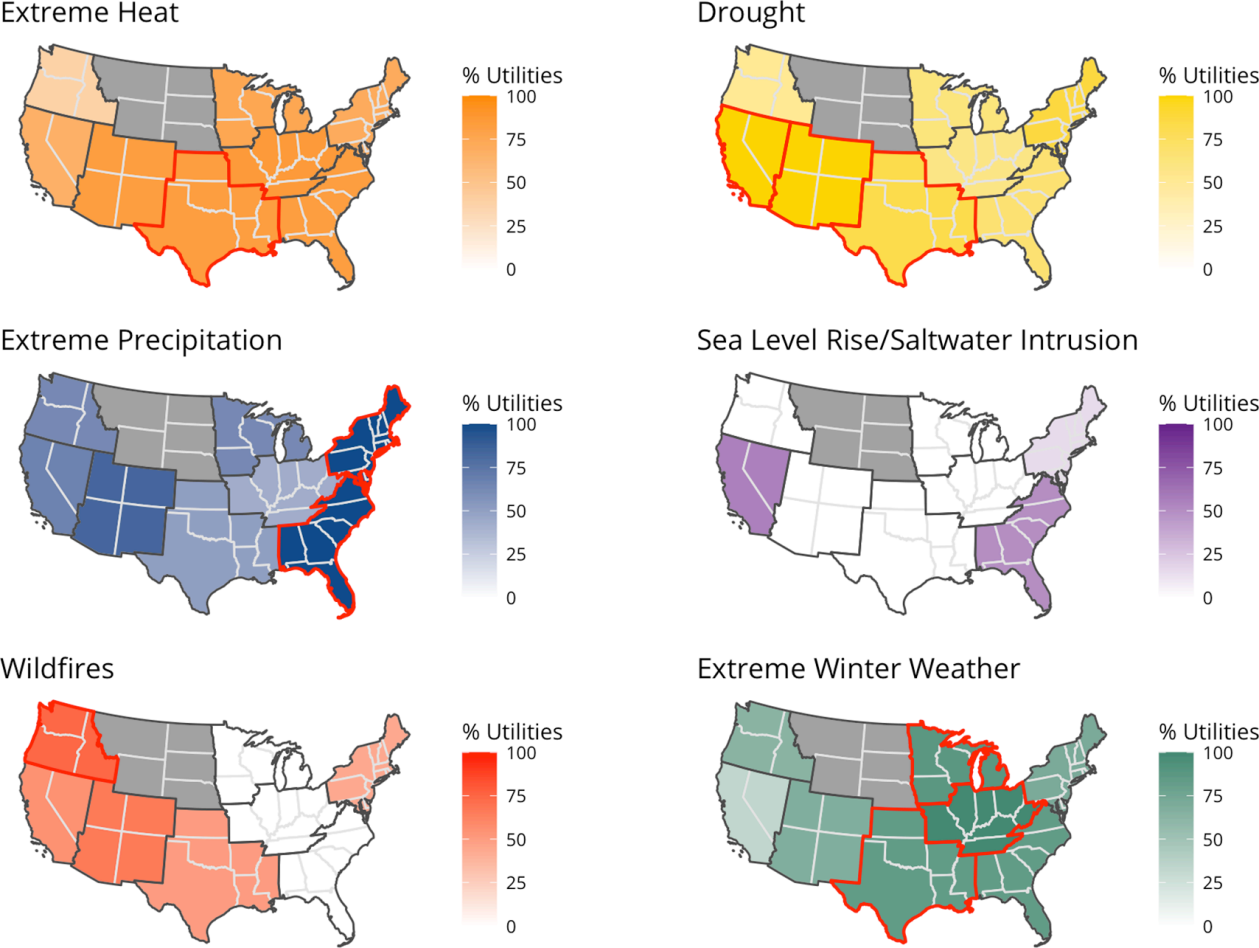
Frequency of identified natural hazard risks to system reliability. Distribution of hazard risks identified by participants in the Continental US, with darker shading indicating a higher percentage of utilities within each region mentioning the risk. Red outlines indicate a region where the specific hazard was identified by the most participants as a risk (i.e., “top” risk in each region). Drought, extreme heat, and extreme cold were equally frequently mentioned by participants from the South region. Northern Rockies and Plains region is not included due to limited sample size. Extreme precipitation includes hurricane and tropical storm or flooding events. Extreme winter weather includes extreme cold events or ice storms/blizzards.

Given the known regional differences in natural hazards and disasters,^62^ participants in different regions expectedly identified different hazards as concerns, demonstrating construct validity.^63^Figure 3 shows the regional distribution of hazards participants identified as a risk to their utility. See SI Figure S3 for a comparison of regional frequencies to the overall frequency.

Participants from the Upper Midwest and Ohio Valley were concerned about extreme cold and ice storms/blizzards affecting system outages, tied to electricity outages and maintenance needs. Comparatively, drought, primarily linked to customer dissatisfaction, was the most frequently mentioned risk for participants from the West and Southwest. All of the Northeast participants identified extreme rainfall as a risk, largely linked to water quality and maintenance needs. Participants in the South expressed concerns about most hazards (except sea level rise and saltwater intrusion). While the region does not experience many extreme cold weather events, Winter Storm Uri (February 2021) was mentioned as reframing utility’s perspectives of their exposure to cold weather challenges. This may represent an example of availability bias or the heuristic that people think “top of mind” considerations first.^64^ News stories about the Western US drought were prevalent when interviews were conducted, which could have additionally introduced availability bias. Some hazards were mentioned by every participant in a region: drought in the West and Southwest, excessive rainfall in the Northeast, hurricanes and tropical storms in the Southeast, and extreme cold events in the Ohio Valley. Regional hazard-reliability matrixes are shown in SI Figures S4–S11.

Although the question was posed to elicit predictions of hazards that could pose risks in the near future, many utilities have used historical experiences to ground their responses. For instance, participants indicated that never having a wildfire or hurricane in their region meant those hazards were not of concern. This aligns with typical design activities, which are based on historical considerations.^1^ Research has found that severe weather concerns are more predictive of water safety concerns than climate change concerns^65^ and that adaptation efforts of wastewater utilities are motivated by improving resilience to past extreme events, not by addressing future climate change.^66^ Use of historical events by participants to describe future risk could suggest a disconnect between risk awareness of current climate hazards and how future changes will affect those hazards.

### 3.3 Developing a Mental Model of Climate-Induced Changes to System Reliability

The five main utility functions that emerged from the coding analysis as being affected by climate change were water supply, water quality, operations and maintenance, business functions, and infrastructure/built components. See SI Table S3 for a frequency table of all emergent codes. All but two participants (97%) mentioned some influence climate change could have on a drinking water utility. Changes to water supply were the most frequently mentioned consequence of climate change; 41 participants (68%) drew links between climate change and water resources. Thirty-two participants (53%) said climate change posed a risk to infrastructure component reliability, either in terms of the lifetime expectancy of certain components or in terms of the engineering factors. Operations and maintenance, business functions, and water quality functions were each mentioned by smaller fractions of participants (23%, 20%, and 15%, respectively). This aligns with previous findings that utility managers struggle to incorporate climate change effects into day-to-day decision-making.^67^ In examining the participants’ utility population served, source water, and geographical location, there are consistent trends in frequency of mentions of each function; a majority of participants in each category are concerned about effects to supply. There are no clear trends for whether population size and source water influence how utilities evaluate climate hazards differently. In terms of job discipline, of nine operations and maintenance directors, none mentioned effects to operations or maintenance needs. Only five of the general directors/managers (20%) mentioned business functions, presumably their primary job focus. Only half of the participants within the engineering discipline discussed effects to infrastructure component reliability. SI Figures S12–S15 present utility function frequency mentions across utility and participant characteristics.

**Figure 4:**
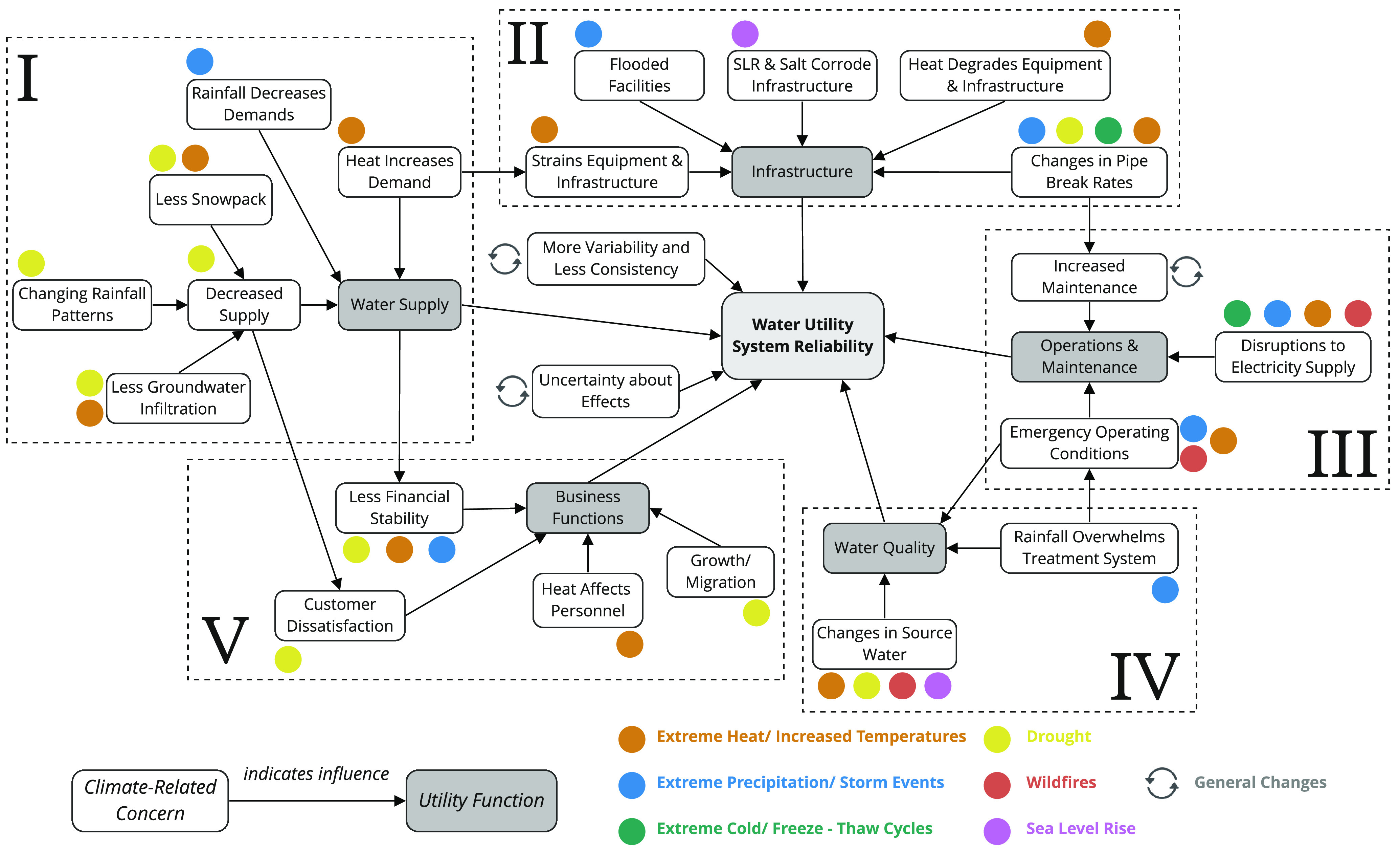
Mental model of climate-induced changes in system reliability.

An influence diagram format was chosen to display the mental model, as it captures the dependencies between variables (arrows indicate influence). The model’s output, shown in the center, is *Water Utility System Reliability*, with the five utility functions as inputs. The emergent codes are shown as the nodes in this model diagram. The primary climate hazards discussed were drought, increased temperatures, and rainfall intensity, with sea level rise, wildfires, and freeze–thaw cycles being mentioned less frequently, likely as they affect fewer utilities. Hazards were typically mentioned individually, although heat and drought were sometimes linked. In contrast to previous work, these interviews revealed that utility managers understand the complexity of climate change effects to the water cycle and extreme weather.^68^ General climate-induced changes (shown as gray circle arrow icon) were identified as introducing more variability and less consistency; there is an understanding that there is still some uncertainty about climate effects. Figure 4 shows the completed water utility mental model, aggregated from all 60 interviews. No participant mentioned all of the nodes or links.

#### 3.3.1 Water Supply

Starting with the most mentioned factor influencing climate-induced changes to system reliability, water supply is shown in Section I (Figure 4), with participants identifying drought (yellow), excessive precipitation (blue), and extreme heat (orange) as climate hazards that affect water resource availability and quantity. Specific effects of these changes were described and are shown as climate-related concerns in the white boxes. For example, changing rainfall patterns and less snowpack, caused respectively by excessive precipitation and drought, are expected to reduce water supplies. Warmer temperatures simultaneously increase demand for water (shown above the water supply box) and reduce groundwater infiltration rates (far left). Participants in all regions identified changes in water resources as a potential consequence of climate change. Those in drier regions noted that climate change could exacerbate the existing supply challenges. One participant mentioned, “We’re in a part of the world where rainfall is hard to come by sometimes. I think we’ve done a great job of drastically improving our reliability and drought resilience, but if our rainfall patterns change drastically and if our rate of evaporation changes drastically over time, obviously we’re going to be much more at risk than we thought we were going to be.” Participants in less drought-prone regions also recognize links between climate change and supply changes; one participant said, “Our watershed is changing... The heavy rains are heavier. The dry periods between these two periods can be drier. Last year we had our first drought; we hadn’t had one in 30 years. It didn’t make the headlines like it did out West, but we did have to implement watering restrictions.” Participants in water-rich areas noted that increases in rainfall in turn can reduce water demand, specifically for irrigation purposes (top left).

#### 3.3.2 Infrastructure

Effects on infrastructure, shown in Section II (Figure 4), were linked to water supply, as the increased demand from extreme heat can strain equipment. For instance, one participant said, “the more you put the squeeze on the water supply, the harder people must work to treat that water to get that water to where they are, which means that there’s an enhanced push on the supply chain. Your equipment’s working harder, your motors aren’t gonna last as long, your pumps aren’t gonna last as long... I see it affecting all of our world, because the increased demand is just gonna put that extra pressure on everything else.” Another identified effect to infrastructure was the failure of electronic or mechanical equipment due to heat degradation and the associated need to maintain air cooling systems. Flooding was linked to pipe washouts and limited access to facilities like pump stations (top left of Section II). Sea level rise and saltwater intrusion (purple) were linked to the degradation of groundwater wells and other buried infrastructure. More generally, extreme conditions (cold, heat, dry soils, or flooding) increase pipe break rates and can shorten infrastructure lifespans. Extreme cold and changes in freeze–thaw cycles are represented in green. Multiple participants noted that these climate hazards would likely exacerbate problems facing already aging infrastructure^69^ and require increased maintenance efforts; some discussed switching from a reactive to a preventative maintenance mindset and embracing asset management.

#### 3.3.3 Operations Maintenance

Participants identified climate change effects on operations and maintenance, shown in Section III (Figure 4). One climate-related concern for operations and maintenance was disruptions to the electricity supply (rightmost white box), which could be caused by winter weather, an extreme storm, extreme heat, or wildfires (red). More broadly, participants noted that heat, wildfires, or extreme storms would require emergency operations. An extreme rainfall event could overwhelm system capacity and affect water treatment plant operations (bottom right). One participant stated that, “power is kind of our biggest concern I would say, because we don’t have a lot of redundancy at our treatment plants.” Another posited that climate change would push the utility into emergency operating conditions, noting, “There’s going to be more times when we’re going to be operating or trying to operate under conditions that are outside of our normal range.” When an extreme event disrupts electricity or source water, utility operators rely on emergency response plans for a course of action. One participant referred to his team’s response to a wildfire event that disrupted water quality as “operational gymnastics.”

#### 3.3.4 Water Quality

In addition to wildfires, participants identified drought, extreme heat, sea level rise/saltwater intrusion, and excessive rainfall events as climate hazards affecting water quality (Section IV of Figure 4). Longer-term disruptions to source water include algal blooms and invasive species linked to hotter temperatures and sea level intrusion into groundwater wells. Excessive rainfall events that overwhelm treatment plant capacity were again linked to the need to have emergency operating conditions. Participants had extensive operational knowledge about how to respond to certain conditions because of experience with historical events and incidents, which aligns with findings that past disasters affect utility manager emergency response actions.^70^ For instance, one participant said, “If the water quality is different as a result of climate change, the makeup of the water could be different... I mean it makes me think about the Flint, Michigan issues. To simplify it, they changed their source water... If the water quality changes, it is going to make a difference inside those pipes. So yeah, the infrastructure could be impacted. Again, it is something that needs to be monitored constantly. The water quality needs to be monitored and tested, and if there are changes, the utility needs to stay on top of those and determine how impactful those changes are and if anything needs to be done.” The continual need for maintenance and monitoring was described as mitigating some potential climate effects. Notable here is how the utility manager thinks about the cascading consequences of climate hazards; changes in demand and source water influence how infrastructure is being used and, in turn, how the utility conducts operations and maintenance.

#### 3.3.5 Business Functions

Finally, participants identified climate-induced changes to business functions, shown in Section V (Figure 4). Cascading changes in water resources, linked to extreme heat, drought, and extreme precipitation, were identified as causing financial instability (see white box linking gray water supply box and gray business functions box). Excess natural supply means the utility could receive less revenue from watering lawns and irrigation. Too little water means the utility must encourage conservation measures and take in less revenue. Effects to business functions were also linked to effects to water supply as participants anticipated that fewer water resources would cause customer dissatisfaction (bottom left). Another identified cascading effect was climate migration and growth (bottom center); utilities in the Great Lakes region and Eastern US saw drought conditions in the Western US as motivating migration into their service areas. Participants noted the challenge of financial planning for those indirect changes. For example, one participant said, “We’re in the Midwest and with so many issues further West with water supply, some of those larger heavy water users think that the Midwest is a great place to land. But it gets challenging and it’s becoming more and more expensive to build treatment capacity.” The other effect on business functions was mentions of heat stress on personnel. The only two participants that mentioned this possible effect were located in hotter areas; they both noted that their utilities already had institutional knowledge about heat stress.

### 3.4 Gaps in Utility Manager Mental Model

The presented expert mental model represents an aggregated descriptive view of how water utility managers understand climate-induced changes to system reliability. However, from an analysis perspective, there are gaps in this understanding. Participants were primarily focused on changes in water resources and indirect consequences to infrastructure rather than direct effects to infrastructure and business functions. These gaps are discussed and contextualized within the existing literature in SI Section 5.

### 3.5 Status of Climate Change Planning Efforts

#### 3.5.1 Beliefs about Climate Change and Its Effects

This work also sought to understand the status of climate resilience and adaptation planning efforts. Participants offered a range of comments about the concept of climate change. Existing work has found misconceptions and omissions in mental models of climate change,^36,39^ as well as misconceptions about its causes and uncertainties about the effectiveness of risk mitigation strategies.^71^ Eight interviewees expressed doubts that climate change was happening and uncertainty about its effects. Some of those participants mentioned natural cycles, overall uncertainties, and debates surrounding climate change (SI Table S4).

There also emerged beliefs about whether climate change would affect a participant’s utility. Eighteen percent (11 out of 60) of participants said they did not believe climate change would influence their water utility system’s overall reliability, and 38% (23 out of 60) of participants said they did not believe climate change would affect their system’s infrastructure. These findings are notable given the availability of literature describing effects of climate change on water utilities and tools designed for these systems to assess their climate risk,^3,4,19−24^ as well as the extensive relationships participants drew between climate hazards and system reliability (shown in Figure 4). Previous work found French water managers perceive climate change at different temporal and spatial scales than they view their water management work;^67^ participants interviewed in this study might have similar disconnects between familiar confidence in their systems’ reliability and the novel risks climate change presents. A small segment of participants (8%, 5 out of 60) believed their existing resilience measures mitigated any potential climate change risk. For instance, one participant said, “It is predicted the events will be more extreme, but we’re getting smarter.” We can analyze participants by utility source water and geographical location to explore trends in the frequency of belief mentions. A majority of Northwest and Upper Midwest region participants said they did not think climate change would affect infrastructure reliability. This aligns with the reduced climate risk faced by these regions. Similarly, groundwater systems and systems that purchase surface water have stronger beliefs about climate change not affecting infrastructure, though trends in beliefs about effects to system reliability are less clear. A higher percentage of utility engineering managers, compared with other disciplines, believe climate change poses no impact to infrastructure functionality. SI Figures S16–S19 report belief frequencies.

#### 3.5.2 Climate Resilience/Adaptation Planning

Regulatory agencies are increasingly shifting focus to require resilience considerations; climate resilience involves the ability to prepare for, recover from, and adapt to multiple changing climate hazards.^72^ The American Water Infrastructure Act of 2018^80^ (AWIA) requires water utilities to identify vulnerabilities to natural hazards and malevolent attacks but does not explicitly require climate change considerations.^73^ Climate risks are recommended considerations when funding water infrastructure projects;^74^ the Infrastructure Investment and Jobs Act^81^ provides funding for specific climate resilience and adaptation projects.^75^

Multiple activities are focused on the support for water resiliency planning. The Water Utility Climate Alliance (WUCA) argues utilities should consider direct links between climate hazards and business functions^4^ and offers recommendations and planning frameworks for how utilities can integrate climate resilience.^3,4^ AWWA recently published a guidance manual on water utility climate action plans.^76^ The EPA’s Creating Water Resilience Utilities division promotes climate resilience planning tools, including the Climate Resilience Evaluation and Awareness Tool and the Vulnerability-Self Assessment Tool, as well as promotes case studies.^22,23^

Even with these federal and professional planning resources, most interviewed water utilities had no formal climate resilience or adaptation plan in place (SI Figures S20–S21). The only utilities interviewed with climate adaptation plans (10%) were from California. Forty percent of participants said their utility did not have any climate-focused resilience or adaptation plan, and 30% of participants said their utility only had a climate-adjacent planning document, such as a Drought Contingency Plan or Emergency Response Plan, which did not make use of future climate projections. Seventeen percent of participants said their municipality did have a climate adaptation plan; however, those plans were focused more on citywide resiliency and less on utility functions. Two combined water and electric utilities reported having climate mitigation plans that were concerned with reducing emissions. Two additional participants referenced climate mitigation efforts, including electrifying vehicle fleets and reducing fossil fuel usage. This is important as climate change will increase energy requirements of utilities across the country.^20^ Though many utilities did not have current climate adaptation plans, 22% of participants indicated that their utilities were at various stages of discussing potential development of such a plan. Previous work found that utility professionals are generally interested in integrating climate projections into utility functions beyond water supply planning.^77^ Participants described technical and institutional limitations for why adaptation planning was not occurring, with institutional barriers mentioned more frequently (SI Section 6 discusses these limitations). Additionally, participants identified other pressing matters as taking precedence over climate adaptation planning; participants mentioned supply chain (17%), staffing issues (13%), and population growth (18%) as affecting water utility system reliability and consuming utility management efforts. To help overcome institutional barriers and concentrate planning efforts, water utilities, like other infrastructure agencies, need more adaptive governance and leadership to maintain service reliability under climate change,^78^ as well as tools to integrate climate planning with other issues.

#### 3.5.3 Belief-Based Categories of Climate Evaluation Efforts

Given the range of concern about climate change and status of planning and adaptation efforts, different classifications can be postulated, based on the differing beliefs and actions. It appears practitioners have different beliefs about whether climate change is happening and which utility functions it will affect, as well as different levels of action on climate-related resilience planning (Table 1). The first category of utilities included participants who do not believe climate change is happening or affecting their utility. This group may have some level of general resilience measures in place from AWIA. The second category is utilities that believe climate change is happening and recognize the risk but think there will be minimal effect to their system, because they are not exposed or have resilience measures in place. Those utilities did not discuss specific risks to infrastructure. The third category expands upon Category 2 and includes utilities actively concerned about climate change, especially risks to water resources, and considering increasing climate resilience of their systems. This might involve including safety factors in the designs or diversifying water supplies. The last category includes utilities that are actively planning for climate-induced changes in water supply, water quality, and infrastructure. This small group mentioned things like scenario planning or some of the discussed tools and resources and exhibited aspects of adaptive management.^79^ Most participants fall into Categories 2 and 3, suggesting directed guidance is needed to improve the state of water utility preparedness.

**Table 1:** Categories of Drinking Water Utility Climate Evaluation Efforts

Category	Coded Beliefs about Climate Change	Climate Adaptation Actions	Number of Utilities
Category 1	Unsure or uncertain about effects of climate change and corresponding hazards	No climate-specific resilience or adaptation planning efforts	8 out of 60 participants
Category 2	Believes climate change is happening, recognizes climate hazards but does not believe they pose risks to either utility infrastructure assets or system reliability	No utility-specific climate adaptation plan, some efforts to increase overall system resilience	22 out of 60 participants
Category 3	Concerned about climate change hazards and believes they could pose a risk to utility functions	No utility-specific climate adaptation plan, some efforts to increase overall system resilience	24 out of 60 participants
Category 4	Believes climate change will affect multiple utility functions, including supply infrastructure, operations and maintenance, water quality, and business functions	Utility-specific adaptation plan, could include specific risk decision-making and/or scenario planning	6 out of 60 participants

## 4 Conclusions

This work developed a mental model of how drinking water utility managers perceive climate change effects to system reliability and offers insights for policy makers to set and implement climate-related strategies within the drinking water sector. This study contributes to the literature by revealing gaps between prescriptive recommendations for climate planning and the descriptive reality of how utility managers consider the climate risks facing their systems. The developed model captures potential climate-induced changes affecting a general large-/medium-sized utility system’s reliability, regardless of geographical location, and can be used by practitioners to understand the scope of potential climate hazards affecting their systems. Specific exposure and vulnerability to climate change hazards determine the individual utility risk. The mental model illustrates current awareness of climate risk and not the level of concern or magnitude of risk.

The interviewed utility managers recognize that climate hazards have the potential to influence utility functions and components of system reliability but may not be concerned about the full scope of their climate risk. The participants were predominately concerned with drought and water supply changes and less aware of effects on operations, maintenance, and finances. The lower frequency of concern for effects on business functions contrasts with the recommendations by WUCA to map climate exposure to business functions. This could indicate an overemphasis of addressing familiar issues (i.e., supply issues) at the harm of leaving the utility vulnerable to emergent issues (i.e., climate risks to finances), potentially due to a lack of knowledge about these emergent risks. When assessing climate-related risks, utilities recognize current hazards and reference historical events and incidents but lack awareness that climate change can exacerbate existing hazards and introduce new hazards.

Despite federal and professional recommendations, few interviewed utility managers are integrating climate projections into planning efforts; there appears to be a lack of climate-focused adaptation and resilience planning taking place. This could be due in part to the confidence interviewed managers had that their systems might not experience changes in reliability due to climate change, or it could be to prioritization of other planning needs. The gaps identified in the mental model (SI Section 5) and the theorized categories of drinking water utility climate evaluation efforts should be the focus of policy interventions; utilities must understand all potential links between climate hazards and utility functions to successfully develop robust adaptation plans. One approach to increasing this understanding could be requiring climate hazards and projections to be considered as part of the mandated AWIA Risk & Resilience Assessments. This would ensure the swath of potential effects of climate hazards on utility functions would be presented to utility management in a familiar manner. In additional to educating utility managers about the nature of these risks, future work should focus on quantifying how climate hazards affect utility business and operations and associated costs; this could allow managers to evaluate and compare the scope of their system’s specific climate risk.

## Supporting Information Available

Additional information about effects of climate change hazards on water utility systems; interview development and protocol; coding dictionary and frequency counts; regional hazard-reliability matrices; additional analysis of codes using participant and utility characteristics; regional distribution of climate adaptation planning status; discussion of gaps in mental model of climate-induced changes to water utility system reliability; discussion of technical and institutional limitations to adaptation planning, as identified by participants (PDF)
