# Supplementary material for: Drinking Water Utility-Level Understanding of Climate Change Effects to System Reliability
Source: ACS ES T Water. 2023 Jul 13;3(8):2395–406. doi: 10.1021/acsestwater.3c00091 (PMC10426323; doi:10.1021/acsestwater.3c00091)
Supplement: Supplementary file 1 — ew3c00091_si_001.pdf [file ew3c00091_si_001.pdf]

Supplemental Information

Title: *Drinking Water Utility-Level Understanding of Climate Change Effects to System Reliability*

Authors

Zia J. Lyle †, ‡; *Jeanne M. VanBriesen* †, ‡; *Constantine Samaras* †, ‡

† Department of Civil and Environmental Engineering, Carnegie Mellon University, 5000 Forbes Avenue, Pittsburgh, Pennsylvania, 15213, United States

‡ Department of Engineering and Public Policy, Carnegie Mellon University, 5000 Forbes Avenue, Pittsburgh, Pennsylvania, 15213, United States

Corresponding author. Zia J. Lyle (email: zlyle@andrew.cmu.edu)

ORCID

Zia J. Lyle: 0000-0001-6368-6765

Jeanne VanBriesen: 0000-0002-2631-0213

Constantine Samaras: 0000-0002-8803-2845

29 Pages

6 Tables

21 Figures

1. Effects of Climate Change Hazards on Water Utility Systems

Table S1. Direct Effects of Climate Change Hazards on Drinking Water Utility System Infrastructure and Water Resources, adopted from[1]

Climate Hazard	Direct Effect to Water Utility System
Drought	Decreased amount of fresh water
Sea Level Rise	Saltwater Intrusion<br>Contamination from pipe corrosion<br>Changes in utility gradient causing pipe fracture<br>Corrosion and degradation of pipes
Increased Temperatures	Pump overheating<br>Pipe corrosion and degradation<br>Increased water demand<br>Growth of pathogens<br>Residual disinfectant decay
Freeze- Thaw Cycles	Underground pipe fracture
Extreme Precipitation Events	Soil expansion and underground pipe cracking<br>Canal breach<br>Bulk water high turbidity and low pH<br>Infrastructure and facility flooding and damage
Wildfires	Bulk water high turbidity and low pH<br>Melting of exposed pump systems and pipe

Detailed Description of Potential Effects of Climate Hazards on Water Utilities

Increased temperatures gradually degrade individual infrastructure components. Electronic/motor overheating failures of pumping and lift stations, which have been linked to service outages during summer heat waves, are expected to increase as ambient temperatures rise.[2,3] Corrosion of metal pipes and degradation of thermoplastic pipes are temperature-dependent; changes in temperature will shorten expected pipe lifespans.[4–7] Climate change may lead to differential soil settlements, as severe heat waves and long droughts lower ground water levels and consolidate soils, increasing damage to underground pipe infrastructure.[8,9]

Extreme precipitation events may compromise functionality and access to infrastructure components. The combination of longer, drier periods followed by periods of extreme rainfall causes soil expansion and settlement; the resulting differential pressure causes pipe cracking and failure.[10,11] Extreme precipitation additionally causes flooding; flooded treatment plants reduce ability to treat drinking water while flooded pump stations limit distribution of clean water.[2]

A climate hazard more specific to coastal utilities is sea level rise, which can also induce flooding, change soil pressure and settlement, and in turn, fracture pipes.[12,13] Increased corrosion rates from saltwater intrusion degrade buried pipe mechanical properties.[14] Saltwater intrusion poses additional risks to water quality throughout the water system.[15]

Another climate hazard, wildfires, can also damage infrastructure and deteriorate water quality. Wildfires induced melting and damage to pipes, as well as other infrastructure components like hydrants valves and meters.[16] Apart from affecting source water quality, wildfires can introduce contamination directly into distribution system water quality because of thermal degradation of pipes, especially PVC pipes.[17,18]

Finally, freeze-thaw cycles have known correlations with pipe breaks.[19,20] Climate change will likely decrease total snowfall in the winter but increase freeze-thaw cycles, though the extent to which that will happen varies by region. In some locations with seasonal cold weather, pipe failure rates might decrease as a result of climate change due to fewer freeze-thaw cycles.[21]

The direct effects of increased temperatures, sea level rise, extreme precipitation, wildfires, and changes in freeze-thaw cycles will decrease individual water infrastructure component reliability, which in turn indirectly affects water utility business functions. These business functions include operations, maintenance, financials, engineering/design, and external

affairs.[22] Climate-induced changes to pipe and pump station lifetimes will require water utilities to change maintenance and operations schedules. More broadly, changes in water quantity and quality will indirectly affect financial viability.

In addition to reducing the reliability of individual infrastructure components and indirectly affecting operations and financials, climate hazards directly affect water utility business functions. As an example, extreme heat affects a utility's ability to conduct maintenance and repairs and requires changes to public communication and external affairs, expanding the utility service mission from just delivery of clean water to communication about reliability.[23] Additionally, climate risk is analyzed when reviewing a water utility's operational risk management and financial management, and may begin to factor into credit and bond ratings.[24] The Water Utility Climate Alliance emphasizes the connection between direct effects to infrastructure and direct and indirect effects to operations and financials, arguing for links between climate risks and water utility business functions.[22] These links are needed to understand how climate change will affect the water utility as an institution, not just effects to individual infrastructure components.

The direct effects of five climate hazards (increased temperatures, sea level rise, extreme precipitation, wildfires, and changes in freeze-thaw cycles) to water infrastructure components were compiled to develop an initial model of climate risks to water utility reliability (Figure S1). This proposed influence diagram illustrates the interconnected nature of water utility reliability and its links to climate hazards; this diagram shows how different climate hazards might affect reliability of different infrastructure components and water utility business functions.

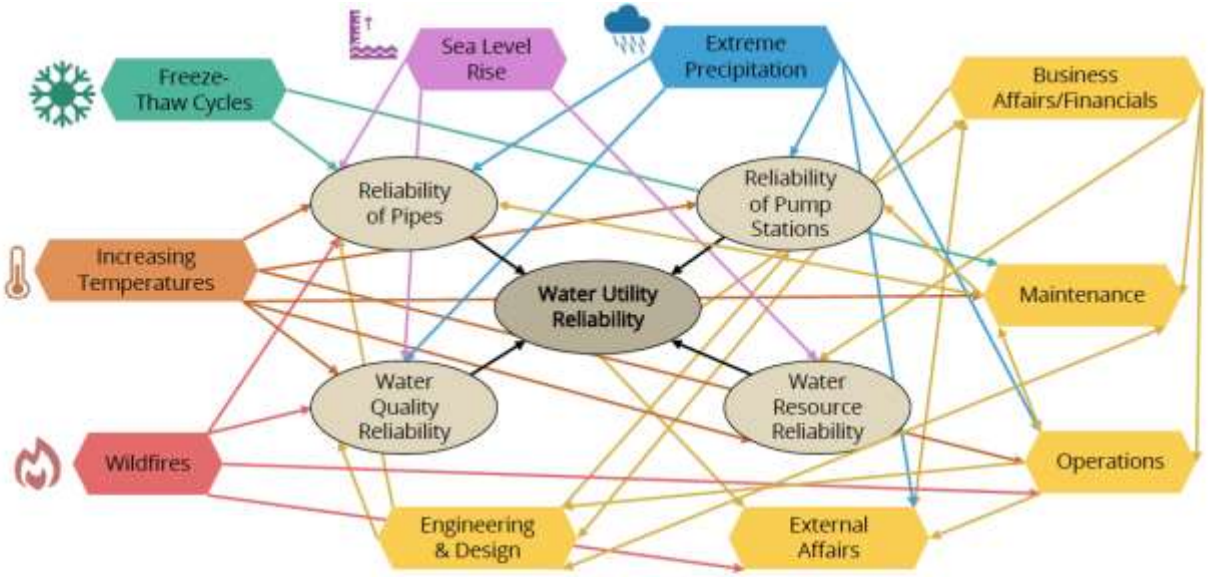

Figure S1. Proposed Water Utility Reliability Influence Diagram

2. Interview Protocol

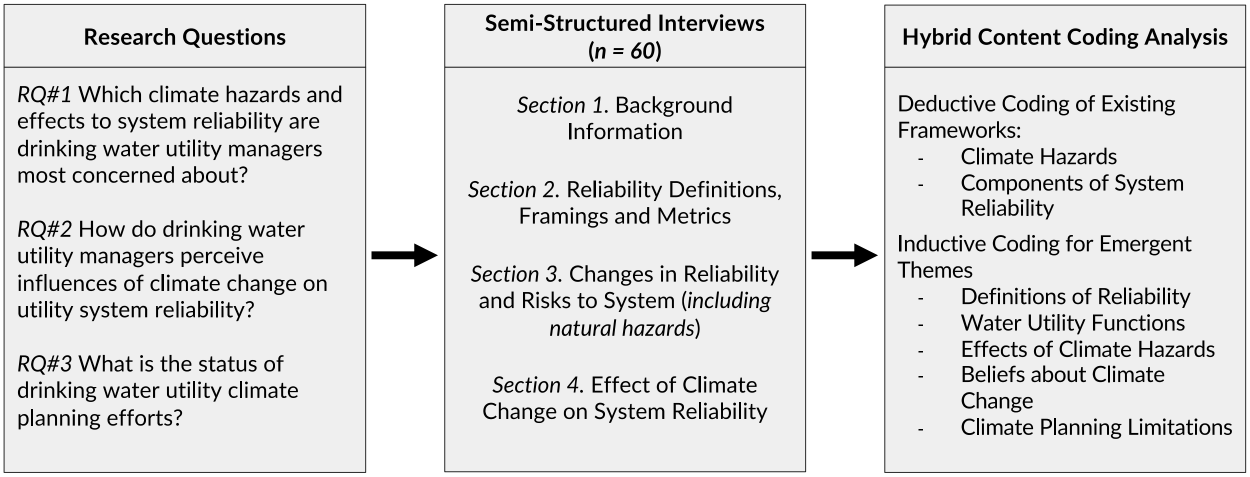

Figure S2. Research Design Summary

The interview begins with questions about the interviewee's background and experience at the utility (Section I). Though drinking water utilities are considered reliable, there is a lack of consistency in reliability of service standards.[25] Section II assess how the interviewee frames "reliability," from particular metrics to understandings in relation to business functions. Specific water utility business functions were compiled[22] and asked about in Question 8. The Environmental Protection Agency tracks water quality compliance violations but not level of service metrics, like length or frequency of outages. There are a range of indicators used to benchmark water utilities, including service outages, maintenance needs, and customer satisfaction indices, but most are self-reported to professional organizations[26] and few are regulated by state public utility commissions.[27] These various reliability indicators were specifically asked about in Question 12. Section III captures how climate risks might change utility reliability. The initial influence diagram (see SI Figure 1) was used to inform which specific events/hazards and reliability metrics were asked about in Question 19. Section IV finally asks specifically about climate change and the status of adaptation planning. Structure of interview questions was informed by work concerning electric utility resilience.[28]

Interview Protocol

Let's first start with some basic information:

Section I: Background

1. Tell me about your water utility, such as system properties and customers served.
2. Tell me about your job. How long have you been working with this water utility and what are your roles and responsibilities?
3. Please walk me through the process your utility has when deciding on new projects. These projects could be part of a capital improvement plan or maintenance/operations decisions.

Now let's discuss the reliability of your drinking water system and water utility:

Section II: Reliability Overview

4. How do you define reliability in relation to your work?
5. Do you think your definition of reliability is influenced by your background & field?
6. In general, are you more concerned about long-term or short-term reliability?
7. Do you think about component reliability (ability of one infrastructure component to function) differently than system reliability (ability of system to deliver clean water)?
8. Which of the following water and non-water business functions do you think influences system reliability?

**Water Utility Functions**	**Influences System Reliability?**
Water Quality Compliance	
Water Resources/Supply Planning	
Business Affairs, Accounting, and Human Resources	
Planning, Modeling, Forecasting, and Analysis	
External Affairs/Customer Satisfaction	
Engineering, Design and Construction	
Operations and Maintenance	

9. What's a recent project your water utility completed that improved your system's reliability?
10. What is your preferred method for communicating how a project will improve system reliability?
11. Does your water utility have a specific metric/measure for system reliability?
12. Which of the following metrics does your utility use when communicating about reliability?

Metrics	Used to Communicate System Reliability?
Number of Water Quality Compliance Violations	
Number of Water Outages	
Average Length of Water Outages	
Infrastructure Component Redundancy	
Maintenance Need	
Customer Satisfaction<br>(*Please provide specific metric if selected)*	
Other?	

13. Which of the identified metrics do you think is most important?
14. Does your utility have level of service expectations associated with this identified metric?

Now let's discuss how the reliability of your water utility has changed over time:

Section III: Changing Reliability

15. Is your drinking water system more or less reliable today than it was 10 years ago?
16. Which functions/departments have seen the biggest changes in reliability over time?
17. What do you think are some of the drivers behind why/how system reliability is changing?
18. In general, do you think people are talking about the same risks as they were 10 years ago?
19. Which of the following hazards/events do you believe could pose risks to your system reliability in the near future?

	Check all metrics you believe the event/hazard could affect.			
**Event/Hazard**	**System Outages**	**Water Quality Compliance Violations**	**Maintenance Needs**	**Customer Satisfaction**
Extreme Heat Event				
Drought				
Hurricane/Tropical Storm				
Excessive Rainfall Event				
Flooding/Storm Surge Event				

Sea Level Rise/Saltwater Intrusion				
Wildfire				
Extreme Cold Event				
Ice Storm/Blizzard				
Operator Error				

20. Did you include any of these identified risks to system reliability as part of your Risk & Reliability Assessment, required under AWIA Section 2013?

Finally, I'd like to discuss the effect of climate change on your water utility.

Section IV: Climate Change

21. Do you think climate change will affect water utility system reliability?
22. Does your utility have a specific climate resilience/adaptation plan?
    a. When was it last updated?
    b. What is included in this plan? Data sources, projections, etc.

3. Coding Dictionary and Frequency Counts

Table S2. Coding Dictionary for Interview Excerpts

Parent Code	Definition	Interview Excerpt	% of Participants
**Business Functions**	Climate change hazards will reduce financial stability or affect other business functions	"The water consumption and revenues are volatile because it depends on how wet each year is. Is it dry or is it wet? We must build in conservation into our rate structure, and we have to kind of anticipate that."	22.0%
**Infrastructure**	Link between climate change hazards and reliability of infrastructure component	"The higher temperatures and the drought issues are going to cause people to use more water for irrigation, which will cause us to have to pump more, which will cause the mains to run more water, so there certainly is going to be use on the system which long term could cause wear and tear and have us have replace parts more often."	55.9%
**More Variability**	Climate change broadly will introduce more variability to utility components	"I think that's really what we're seeing with the climate change discussion; we're losing that consistency where you're riding along you get a blip up and then, you get a blip down. Now it's like every year we're either really hot, we're really cold, we're really wet, or we're really dry."	5.10%
**No Impact**	Does not believe climate change will affect utility system in any way	"I don't see [climate change] as affecting our system right now or in the near future."	50.8%
**Operations & Maintenance**	Climate change hazards will have consequences for O&M processes	"I think that there's going to be more times when we're going to be operating or trying to operate under conditions that are outside of our normal range."	23.7%
**Supply**	Climate change hazards will affect availability of water resources	"We're thinking that as climate changes take effect, we're gonna have the same amount of water but it's gonna be timed differently. Instead of being snow in the winter, it's gonna be rain in the winter."	69.5%
**Uncertain About Impact**	Believe climate change hazards could affect utility functions but not sure exactly how	"Climate change is kind of that big wildcard like, 'What is it? When is it? For what degree is it?'"	10.2%
**Water Quality**	Connection between climate change hazards and water quality changes	"We are noticing that [climate change] is also driving harmful algal blooms within our source."	15.3%

Table S3. Climate Change Effects to System Reliability: Codes & Sub-codes

Parent Code	Child Codes	Definition	Total Mentions	% of Participants
Business Functions		Climate change hazards will reduce financial stability or affect other business functions	15	22.00%
	Impacts Personnel	Climate change will affect utility employees	2	3.40%
	More Growth/Migration	Climate change will lead to migration or growth within utility service area	4	6.80%
	Negatively Impacts Financials	Climate change will cost the utility or affect revenue	8	13.60%
Infrastructure		Link between climate change hazards and reliability of infrastructure component	46	55.90%
	Changing Demand Strains Infrastructure and Equipment	Climate change will require existing infrastructure or equipment to work more	10	15.30%
	Cold Shortens Lives	Cold extreme weather reduces lifespan of infrastructure or equipment	1	1.70%
	Electronic/Mechanical Equipment Failure	Climate change poses risks to electronic or mechanical equipment	10	16.90%
	Degradation From Heat	Climate change hazard of concern is specifically extreme heat	8	13.60%
	SCADA System	Equipment	1	1.70%
	Extreme Events	Low frequency, high impact events are of a concern to the utilities infrastructure	2	3.40%
	Facility Flooding	Flooding events are identified as posing risks to utility facilities	5	6.80%
	Heat Shortens Lives	Hot extreme weather reduces lifespan of infrastructure or equipment	4	6.80%
	Pipe Breaks	Climate change poses risk to pipe break rates	11	15.30%
	Differential Soil Pressure	Extreme weather causes soil to contract/expand, which affects pipe break rates	1	1.70%
	Pipe Breaks in Cold Weather	Extreme cold weather affects pipe break rates	5	8.50%
	Pipe Washouts	Flooding or excessive rainfall causes pipe breaks	7	8.50%
	Sea Level Rise/Saltwater Intrusion	Salt water intrusion identified as a climate hazard affecting utility infrastructure	6	8.50%
	Groundwater Wells	SLR identified as contaminating groundwater wells	1	1.70%
	Worse Because Of Age	Any consequences of climate hazards are exacerbated by current age of infrastructure	1	1.70%
More Variability		Climate change broadly will introduce more variability to utility components	5	5.10%
No Impact		Does not believe climate change will affect utility system in any way	38	50.80%
	Have Resilience Measures in Place	Indicates existing utility resilience measures will reduce any climate risk	5	8.50%
	No Impact to Infrastructure	Indicates there is no concern that climate change will affect infrastructure component reliability	24	40.70%
	No Impact to System Reliability	Indicates there is no concern that climate change will affect water utility system reliability	12	18.60%
Operations & Maintenance		Climate change hazards will have consequences for O&M processes	18	23.70%
	Changing Maintenance Needs	Climate change hazards will require changes to utility maintenance requirements and processes	2	3.40%
	Disruptions To Electricity Supply	Climate change hazards will disrupt electricity supply to utility	9	10.20%
	Other Utilities	Other utilities are specifically at risk of disruptions to electricity supply	1	1.70%
	Emergency Operations	Utility will have to operate in emergency conditions due to climate change hazards	4	5.10%
	Water Quality Operations	Emergency conditions will specifically related to water quality operations	2	3.40%
Supply		Climate change hazards will affect availability of water resources	62	69.50%
	Droughts Decrease Supply	Drought is a climate hazards that will decrease water supply availability for the utility	37	47.50%
	Less Snowpack	Climate change induced drought will specifically reduce snowpack availability	7	10.20%
	More Evaporation	Climate change induced drought will specifically increase evaporation rates	1	1.70%
	Shifting Rainfall	Climate change induced drought is linked to rainfall shifting elsewhere	6	10.20%
	Worse For Groundwater Systems	Climate change induced drought is a greater risk for groundwater systems	3	5.10%
	Heat Increases Demand	Increased temperatures lead to more demand on water utility	12	16.90%
	Intense Rainfall Overwhelms Treatment Plants	Extreme rainfall events overwhelm treatment plant and reduce utility supply of clean water	4	6.80%
	Rain Decreases Demands	Extreme rainfall events and more precipitation decreases demand for utility supply of clean water	2	3.40%
	Relocating Water	Climate hazards will lead to demands for water in different places, thus shifting location of supply	1	1.70%
Uncertain About Impact		Believe climate change hazards could affect utility functions but not sure exactly how	6	10.20%
Water Quality		Connection between climate change hazards and water quality changes	16	15.30%
	Algal Blooms	Climate change will affect rates of algal blooms	4	3.40%
	Changes In Demand	Climate change induced changes in demand will affect source water quality	1	1.70%
	Invasive Species	Climate change will affect rates of invasive species in supply	3	1.70%
	SLR/Saltwater Intrusion	Salt water intrusion identified as a climate hazard affecting water quality supply	1	1.70%
	Wildfires	Wildfires identified as a climate hazards affecting water quality supply	3	5.10%

Table S4. Climate Change Discourse: Codes & Sub-codes

Parent Code	Definition	Total Mentions	% of Participants
"Climate Change Is Real"	Statement acknowledging there exists climate denialism	5	8.50%
"Unless/If X Event Happens"	Anticipating climate change hazards will likely fall within expected range and there won't be any risk unless this is not the case	1	1.70%
Depends on Water Supply	Climate change risk is linked to utilities water supply	2	1.70%
Hope It Doesn't Happen	"wishing","hoping", emotional response without concrete preparations	3	5.10%
Multiple Hazards at Once	Climate change understood as hazard compounding	1	1.70%
Need To Get Creative	Climate change hazards requires creative solutions	4	5.10%
Need To Be Prepared	Utility needs resilience measures or general preparedness because climate change is a risk	10	15.30%
Political Topic	Climate change is political, has negative connotation	8	11.90%
Public Confidence in Utility	Regardless of climate risk, utility will be prepared	2	1.70%
Public Uninformed	Public doesn't care about climate change or understand the risk	2	3.40%
Uncertainty That It Is Happening	General climate change denialism	4	6.80%
Unsure About Consequences	Recognizes climate change is happening but not sure whether its effects will be significant	4	6.80%
Will Impact Everybody	Climate change will have universal impact, no need to have unique plan	3	5.10%
Will Affect Systems Differently	Climate change will affect certain systems more than others, location-specific risks	1	1.70%

4. Additional Results

	All Participants	West (n = 9)	Northwest (n = 8)	Northeast (n = 7)	Southeast (n = 6)	Up. Midwest (n = 8)	South (n = 6)	Ohio Valley (n = 7)	Southwest (n = 6)
Drought	72%	100%	50%	86%	67%	63%	83%	57%	100%
Extreme Heat Event	68%	67%	38%	71%	83%	75%	83%	86%	83%
Extreme Cold Event	67%	33%	50%	71%	83%	88%	83%	100%	67%
Excessive Rainfall Event	58%	67%	50%	100%	50%	63%	50%	29%	83%
Flooding/Storm Surge Event	53%	67%	63%	57%	67%	50%	50%	43%	50%
Ice Storm/Blizzard	48%	0%	63%	57%	67%	38%	83%	86%	33%
Wildfire	35%	56%	75%	43%	0%	0%	50%	0%	67%
Hurricane/Tropical Storm	30%	11%	0%	86%	100%	0%	33%	43%	0%
Sea Level Rise/Saltwater Intrusion	15%	56%	0%	14%	50%	0%	0%	0%	0%

Figure S3. Identified Hazard Risks to System Reliability (n = 60). Hazards ordered in terms of overall concern by study participants; bar graphics and percentages illustrate same information. Northern Rockies and Plains region not included due to limited sample size (n = 3).

	% Utilities Mentioning	System Outages	Water Quality Compliance	Maintenance Needs	Customer Satisfaction
% Utilities Mentioning		*100%*	*78%*	*100%*	*89%*
Extreme Heat Event	*67%*	3	3	6	2
Drought	*100%*	6	6	5	8
Hurricane/Tropical Storm	*11%*	1	1	0	1
Excessive Rainfall Event	*67%*	4	5	3	2
Flooding/Storm Surge Event	*67%*	5	3	4	1
Sea Level Rise/Saltwater Intrusion	*56%*	2	2	3	2
Wildfire	*56%*	5	5	3	3
Extreme Cold Event	*33%*	2	1	3	1
Ice Storm/Blizzard	*0%*	0	0	0	0

Figure S4. Hazard – Reliability Matrix for West Region (n = 9)

	% Utilities Mentioning	System Outages	Water Quality Compliance	Maintenance Needs	Customer Satisfaction
% Utilities Mentioning		*75%*	*88%*	*63%*	*63%*
Extreme Heat Event	*38%*	4	0	3	3
Drought	*50%*	3	1	0	4
Hurricane/Tropical Storm	*0%*	0	0	0	0
Excessive Rainfall Event	*50%*	3	4	1	4
Flooding/Storm Surge Event	*63%*	5	6	3	4
Sea Level Rise/Saltwater Intrusion	*0%*	0	0	0	0
Wildfire	*75%*	5	6	4	3
Extreme Cold Event	*50%*	2	1	4	4
Ice Storm/Blizzard	*63%*	5	2	5	5

Figure S5. Hazard – Reliability Matrix for Northwest Region (n = 8)

	% Utilities Mentioning	System Outages	Water Quality Compliance	Maintenance Needs	Customer Satisfaction
% Utilities Mentioning		*100%*	*100%*	*100%*	*71%*
Extreme Heat Event	*71%*	1	2	2	1
Drought	*86%*	4	3	2	3
Hurricane/Tropical Storm	*86%*	4	5	5	1
Excessive Rainfall Event	*100%*	2	5	4	1
Flooding/Storm Surge Event	*57%*	3	2	2	2
Sea Level Rise/Saltwater Intrusion	*14%*	0	0	1	0
Wildfire	*43%*	1	2	1	2
Extreme Cold Event	*71%*	3	0	5	1
Ice Storm/Blizzard	*57%*	1	1	4	2

Figure S6. Hazard – Reliability Matrix for Northeast Region (n = 7)

	% Utilities Mentioning	System Outages	Water Quality Compliance	Maintenance Needs	Customer Satisfaction
% Utilities Mentioning		*67%*	*67%*	*100%*	*67%*
Extreme Heat Event	*83%*	2	2	2	3
Drought	*67%*	2	2	2	3
Hurricane/Tropical Storm	*100%*	4	2	5	4
Excessive Rainfall Event	*50%*	2	1	3	2
Flooding/Storm Surge Event	*67%*	2	1	4	2
Sea Level Rise/Saltwater Intrusion	*50%*	0	2	2	1
Wildfire	*0%*	0	0	0	0
Extreme Cold Event	*83%*	2	1	5	2
Ice Storm/Blizzard	*67%*	3	2	4	3

Figure S7. Hazard – Reliability Matrix for Southeast Region (n = 6)

	% Utilities Mentioning	System Outages	Water Quality Compliance	Maintenance Needs	Customer Satisfaction
% Utilities Mentioning		*63%*	*63%*	*88%*	*50%*
Extreme Heat Event	*75%*	3	1	6	2
Drought	*63%*	2	2	4	4
Hurricane/Tropical Storm	*0%*	0	0	0	0
Excessive Rainfall Event	*63%*	2	1	4	0
Flooding/Storm Surge Event	*50%*	3	2	5	2
Sea Level Rise/Saltwater Intrusion	*0%*	0	0	0	0
Wildfire	*0%*	0	0	0	0
Extreme Cold Event	*88%*	4	0	8	2
Ice Storm/Blizzard	*38%*	3	1	3	2

Figure S8. Hazard – Reliability Matrix for Upper Midwest Region (n = 8)

	% Utilities Mentioning	System Outages	Water Quality Compliance	Maintenance Needs	Customer Satisfaction
% Utilities Mentioning		*83%*	*100%*	*100%*	*83%*
Extreme Heat Event	*83%*	3	3	5	3
Drought	*83%*	1	2	5	4
Hurricane/Tropical Storm	*33%*	1	2	1	1
Excessive Rainfall Event	*50%*	1	1	2	2
Flooding/Storm Surge Event	*50%*	1	2	3	2
Sea Level Rise/Saltwater Intrusion	*0%*	0	0	0	0
Wildfire	*50%*	2	1	3	2
Extreme Cold Event	*83%*	3	3	5	4
Ice Storm/Blizzard	*83%*	4	3	5	4

Figure S9. Hazard – Reliability Matrix for South Region (n = 6)

	% Utilities Mentioning	System Outages	Water Quality Compliance	Maintenance Needs	Customer Satisfaction
% Utilities Mentioning		*100%*	*86%*	*86%*	*71%*
Extreme Heat Event	*86%*	3	4	3	3
Drought	*57%*	2	1	2	4
Hurricane/Tropical Storm	*43%*	3	1	1	0
Excessive Rainfall Event	*29%*	1	1	1	1
Flooding/Storm Surge Event	*43%*	1	1	2	2
Sea Level Rise/Saltwater Intrusion	*0%*	0	0	0	0
Wildfire	*0%*	0	0	0	0
Extreme Cold Event	*100%*	6	2	6	3
Ice Storm/Blizzard	*86%*	6	2	4	2

Figure S10. Hazard – Reliability Matrix for Ohio Valley Region (n = 7)

	% Utilities Mentioning	System Outages	Water Quality Compliance	Maintenance Needs	Customer Satisfaction
% Utilities Mentioning		100%	67%	100%	100%
Extreme Heat Event	*83%*	3	2	2	2
Drought	*100%*	3	3	3	5
Hurricane/Tropical Storm	*0%*	0	0	0	0
Excessive Rainfall Event	*83%*	4	4	3	2
Flooding/Storm Surge Event	*50%*	3	3	3	2
Sea Level Rise/Saltwater Intrusion	*0%*	0	0	0	0
Wildfire	*67%*	3	2	3	2
Extreme Cold Event	*67%*	3	1	4	3
Ice Storm/Blizzard	*33%*	2	1	1	2

Figure S11. Hazard – Reliability Matrix for Southwest Region (n = 6)

	Overall	10,001-50,000	50,001-100,000	100,001-250,000	250,001-500,000	> 500,000
	n = 60	*n = 5*	*n = 15*	*n = 20*	*n = 9*	*n = 11*
Supply	68%	80%	73%	53%	89%	73%
Infrastructure	53%	40%	60%	42%	56%	73%
O&M	22%	40%	40%	5%	11%	27%
Business Functions	20%	0%	20%	16%	22%	36%
Water Quality	15%	20%	13%	5%	11%	36%

Figure S12. Participants Discussing Climate Change Effects to System Reliability (n=60), divided by utility population served

	Overall	Ground Water	Surface Water	Surface Water - Purchased
	n = 60	*n = 11*	*n = 40*	*n = 9*
Supply	68%	80%	63%	88%
Infrastructure	53%	60%	58%	25%
O&M	23%	20%	23%	25%
Business Functions	20%	20%	20%	25%
Water Quality	15%	30%	15%	0%

Figure S13. Participants Discussing Climate Change Effects to System Reliability (n=60), divided by Source Water Classification

	Overall	Northeast	N. Rockies & Plains	Northwest	Ohio Valley	South	Southeast	Southwest	Up. Midwest	West
	n = 60	*n = 7*	*n = 3*	*n = 8*	*n = 7*	*n = 6*	*n = 6*	*n = 6*	*n = 8*	*n = 9*
Supply	68%	57%	100%	75%	57%	67%	40%	100%	63%	78%
Infrastructure	53%	71%	67%	25%	29%	67%	60%	83%	25%	78%
O&M	23%	14%	33%	25%	14%	17%	0%	17%	50%	22%
Business Functions	20%	14%	0%	13%	29%	33%	0%	33%	25%	22%
Water Quality	15%	14%	33%	0%	14%	0%	20%	0%	25%	33%

Figure S14. Participants Discussing Climate Change Effects to System Reliability (n=60), divided by NOAA Climate Region

	Overall	Asset Management	Director/ Executive	Distribution System	Engineering	Operations & Maintenance	Water Resources	Water Treatment
	n = 60	*n = 2*	*n = 25*	*n = 3*	*n = 14*	*n = 9*	*n = 2*	*n = 5*
Supply	68%	50%	60%	100%	71%	78%	100%	80%
Infrastructure	53%	100%	52%	0%	57%	56%	50%	60%
O&M	23%	100%	36%	0%	14%	0%	50%	0%
Business Functions	20%	50%	24%	0%	7%	22%	50%	20%
Water Quality	15%	0%	8%	0%	21%	11%	0%	60%

Figure S15. Participants Discussing Climate Change Effects to System Reliability (n=60), divided by Job Discipline

	Overall	10,001-50,000	50,001-100,000	100,001-250,000	250,001-500,000	> 500,000
	n = 60	*n = 5*	*n = 15*	*n = 20*	*n = 9*	*n = 11*
No Impact to Infrastructure	38%	20%	47%	42%	44%	27%
No Impact to System Reliability	18%	0%	7%	26%	33%	18%
Have Resilience Measures	8%	20%	47%	42%	44%	27%

Figure S16. Participants Believing No Climate Change Effects to System Reliability (n=60), divided by utility population served

	Overall	Ground Water	Surface Water	Surface Water - Purchased
	n = 60	*n = 11*	*n = 40*	*n = 9*
No Impact to Infrastructure	38%	50%	33%	63%
No Impact to System Reliability	18%	20%	20%	13%
Have Resilience Measures	8%	10%	10%	0%

Figure S17. Participants Believing No Climate Change Effects to System Reliability (n=60), divided by Source Water Classification

	Overall	Northeast	N. Rockies & Plains	Northwest	Ohio Valley	South	Southeast	Southwest	Up. Midwest	West
	n = 60	*n = 7*	*n = 3*	*n = 8*	*n = 7*	*n = 6*	*n = 6*	*n = 6*	*n = 8*	*n = 9*
No Impact to Infrastructure	38%	29%	0%	75%	43%	33%	40%	0%	63%	33%
No Impact to System Reliability	18%	14%	0%	25%	14%	33%	40%	0%	38%	0%
Have Resilience Measures	8%	0%	0%	0%	0%	17%	20%	17%	13%	11%

Figure S18. Participants Believing No Climate Change Effects to System Reliability (n=60), divided by NOAA Climate Region

	Overall	Asset Management	Director/ Executive	Distribution System	Engineering	Operations & Maintenance	Water Resources	Water Treatment
	n = 60	*n = 2*	*n = 25*	*n = 3*	*n = 14*	*n = 9*	*n = 2*	*n = 5*
No Impact to Infrastructure	38%	0%	36%	50%	57%	33%	50%	20%
No Impact to System Reliability	18%	0%	20%	0%	7%	33%	0%	40%
Have Resilience Measures	8%	0%	12%	0%	0%	11%	0%	20%

Figure S19. Participants Believing No Climate Change Effects to System Reliability (n=60), divided by Job Discipline

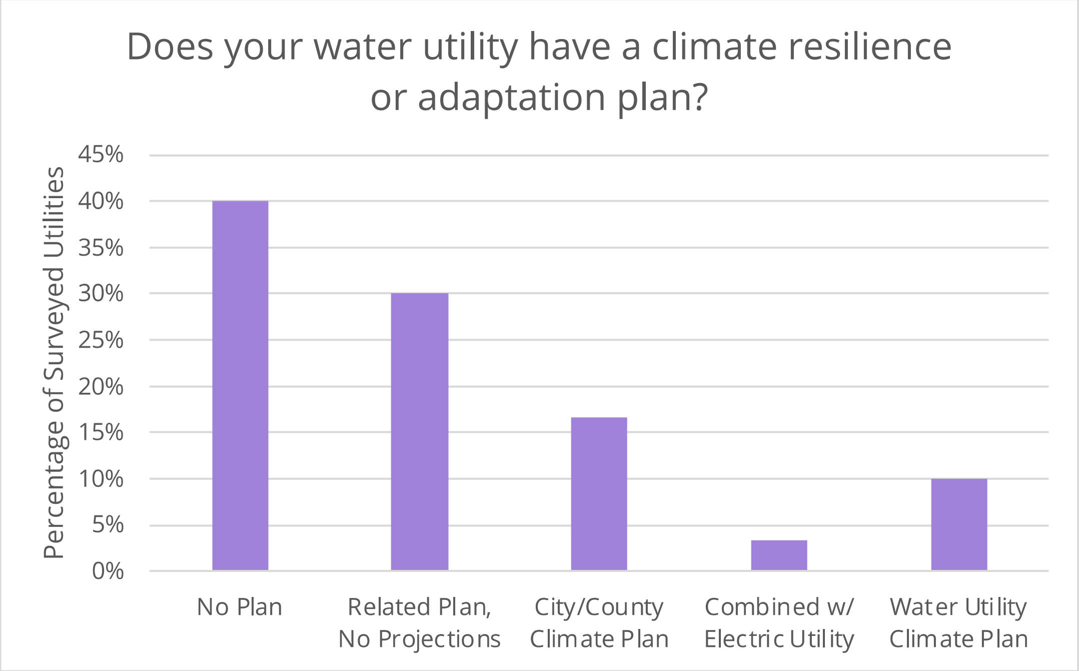

Figure S20. Status of Climate Planning Efforts, n = 60

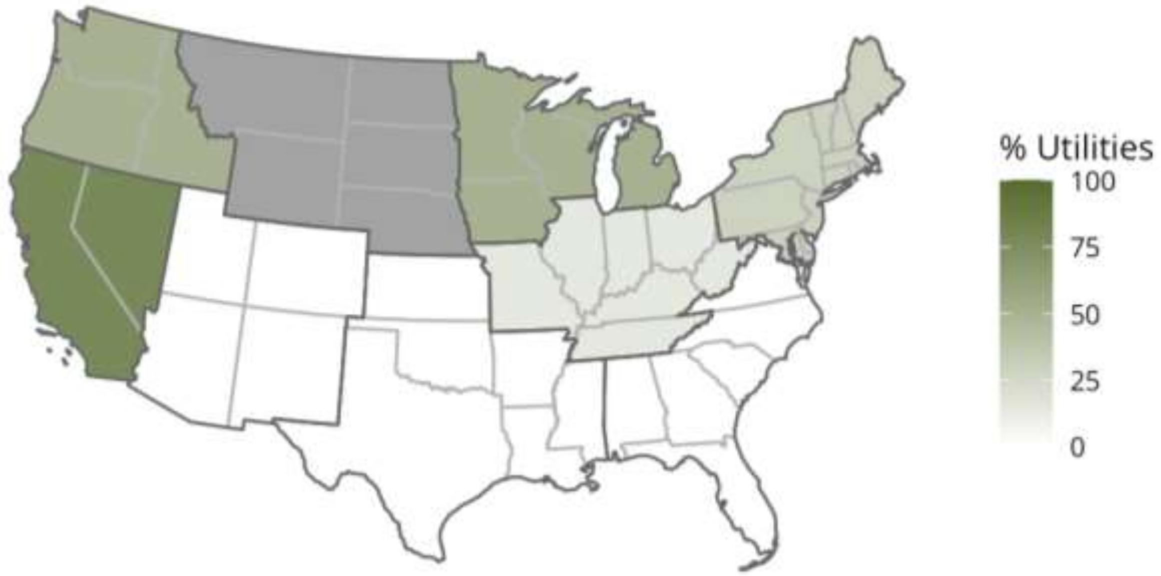

Figure S21. Prevalence of Self-Reported Water Utility Climate Adaptation Plans within Interviewed Participants (n = 60) Darker shading indicates more utilities within each region have a utility-specific or city/county-specific climate adaptation or action plan

5. Gaps in Mental Model

The results indicate that water utility managers' understanding of climate-induced changes to system reliability is primarily focused on changes in water resources and supply. Participants demonstrated understanding of how climate change is expected to affect water resources, but they appear less certain about how other factors will be affected. Some participants shared robust understandings of climate projections in their regions; one stated, "What we see in our area for climate change is that storms in the wintertime will be sort of maybe more prominent, the average rainfall will be similar, but rainfall events and flooding events may be more severe. And with the lack of snowpack, which helps us to restore our aquifers, I do think about the long-term impacts to aquifers." Only four participants referred to the hazard-reliability matrix when asked about climate change and only two participants explicitly identified the hazards in that matrix as associated with climate change. This could suggest a disconnect between risk awareness from current climate hazards and how future changes to those hazards will affect utility risk. Most participants identified an individual hazard (typically drought) as their "relevant" climate hazard and discussed changes in system reliability in relation to that individual hazard, rather than discussing a range of relevant climate hazards.

Participants identified cascading, indirect consequences of climate change hazards to infrastructure (i.e., how heat-related changes in demand will strain pump stations and pipes). Less discussed were the direct effects of climate hazards on infrastructure. Increased temperatures can degrade individual infrastructure components. Only 8 out of 60 participants identified concerns about electronic/motor overheating failures of pumping and lift stations, which have been linked to service outages during summer heat waves[2] and are expected to increase as ambient temperatures rise.[3] There were several mentions of increased temperatures

affecting pipe breaks, but no participants described temperature-dependent corrosion of metal pipes and degradation of thermoplastic pipes, which may shorten pipe lifespans.[4–7] Climate change may lead to differential soil settlements, as severe heat waves and long droughts lower ground water levels and consolidate soils. These changes can damage underground pipe infrastructure.[8,9] Further, the combination of longer, drier periods followed by periods of extreme rainfall can cause soil expansion and settlement; the resulting differential pressure can cause pipe cracking and failure.[10,11] Only one participant mentioned climate change affects to soil properties and buried infrastructure. Five participants noted that the amount of buried infrastructure in a water system provided resilience against hazards.

Extreme precipitation often causes flooding, and flooded treatment plants and pump stations can reduce the utility's ability to treat and distribute drinking water.[2] Of the four participants who mentioned climate-related flooding of facilities, three had experienced large flooding events recently. Coastal utilities mentioned concerns about sea level rise, which can also induce flooding, change soil pressure and settlement, and in turn, fracture pipes.[12,13] Saltwater intrusion degrades buried pipe mechanical properties[14] and poses risks to water quality throughout the water system;[15] five participants discussed this degradation and identified water quality issues with salt water intrusion into groundwater wells.

Another climate hazard, wildfires, can deteriorate water quality, which was mentioned by three participants (all from western regions where wildfire is common). Apart from affecting source water quality, wildfires can introduce contamination directly into distribution system water quality because of thermal degradation of PVC pipes or infrastructure components like hydrants valves and meters.[16,17] Only one participant identified this potential consequence.

Finally, freeze-thaw cycles have known correlations with pipe breaks.[19,20] Climate change will likely decrease total snowfall in the winter but increase freeze-thaw cycles, though the extent to which that will happen varies by region. In some locations with seasonal cold weather, pipe failure rates might decrease as a result of climate change due to fewer freeze-thaw cycles.[21] Five participants from the Upper Midwest and Ohio Valley regions noted that climate change could affect cold temperatures, in turn changing pipe break rates.

In addition to gaps in understanding the potential effects of specific climate hazards on infrastructure, the aggregated mental model also illustrates gaps related to direct effects of increased temperatures, sea level rise, extreme precipitation, wildfires, and changes in freeze-thaw cycles on water utility business functions. As an example, extreme heat affects a utility's ability to conduct maintenance and repairs and requires changes to public communication and external affairs, expanding the utility service mission from delivery of clean water to communication about reliability.[23] Only two participants identified increased heat exposure and stress as potentially affecting personnel. In the participants' responses to the hazard-reliability matrix changes in maintenance need was identified as the most common consequence of the climate hazards, but when asked about effects of climate change, few participants identified changing maintenance needs. On the management side, climate risk is analyzed when reviewing a water utility's operational risk management and financial management, and may begin to factor into credit and bond ratings,[24] but no participants mentioned credit or bond risks from exposure to climate change. Finally, half of the participants identified challenges with communicating with customers, with some expressing that customer perceptions and expectations of the drinking water sector have soured in recent years (five out of 60 participants).

6. Discussion of Institutional and Technical Barriers

Participants described both technical and institutional limitations for why adaptation planning wasn't taking place, though institutional barriers were mentioned more frequently. Technical limitations (**Table S5**) describe barriers to engineering project designs and execution; participants highlighted the uncertainty associated with climate projections, the potential for magnifier events or tipping points, and the expense as reasons for why the utility has not integrated climate adaptation into planning efforts. These technical limitations mirror those identified a decade ago,[29] indicating progress is still needed in providing utilities with usable climate projections they can easily integrate into existing planning processes. Ten out of sixty participants (17%) said their utility used climate change projections in planning efforts; streamflow and rainfall intensity projections were most commonly mentioned, followed by projections of sea level rise. Three participants mentioned using climate projections in the utility's future demand models to assess how climate change affects water consumption and usage. Only one interviewed participant described their utility's use of climate scenario planning to determine what future climate projections to design for; their utility was making decisions about the level of water supply reliability based on different future climate scenarios. Only one participant mentioned any of the EPA's CRWU tools; he identified the CREAT tool as one that his utility hasn't made use of yet but hopes to in the future. Eleven out of the 60 participants (18%) discussed tracking changes to source water, infrastructure assets, and water quality as a way to assess when climate change effects begin to be observed.

Table S5. Technical Limitations to Climate Adaptation Planning Code Frequency

Parent Code	Child Codes	Definition	Total Mentions	% of Participants
Technical Limitations		Things that limit the technical ability to undergoe climate planning		
	Climate Change Can't be Planned For	Climate hazards events are so extreme it is not realistic to design for them	5	8%
	Effects of Climate Change are not Quantifiable	Climate change hazards are observed but can't be quantified yet to incorporate into planning	4	7%
	Climate Changes are Too Gradual	Climate change hazards happen slowly and don't need to be planned for yet	1	2%
	Long Time Horizon	Climate change happens over long time period, making it difficult to plan for	3	5%
	Climate Change has Magnifier Effects	Cascading effects of climate hazards making it difficult to plan for	4	7%
	Non-reversible Tipping Points	Some consequences of climate hazards are large tipping points that can't be planned for	1	2%
	Too expensive/Impractical	Climate planning requires too many resources	5	8%
	Too Uncertain	Uncertainty surrounding climate change reduces ability to develop plans	12	20%

Institution limitations (**Table S6**) include structural barriers within and external to the utility that limit investment of time and planning efforts. Interview participants mentioned climate change as posing a greater risk to wastewater and stormwater systems than drinking water systems, either because excessive rainfall or sea level rise was perceived as affecting those infrastructure systems more. Twenty-five percent of participants (15 out of 60) discussed how climate change was a problem affecting “other” utilities in other places, with nine participants specifically mentioning the Western portion of the United States and four referencing flood-prone areas. For example, one participant said, “There's definitely other parts of the country that [climate adaptation planning] is absolutely critical. And as they get more severe weather events that are taking out critical components of their system, they can't provide water. It’s becoming more likely for an event like that to occur again. But again, we don't see it affecting us as much in this particular region.” As demonstrated in the compiled mental model, participants were aware of the general vulnerabilities to climate change a water utility might face and accurately understood that there are regional differences in changing climate hazards. When asked about the effects of climate change on his utility’s drinking water system, one participant said, “I think in different parts of the country, you’re gonna get different answers. In the part of the country where the utility is water rich, you'll get one answer. If you're out in the arid west, or in

California, you're gonna get a different answer. Those folks are experiencing 20-year droughts and they're gonna be many times more concerned about climate change. And I would say, the more water rich the utility, the less concerned they're gonna be with climate change.” While climate risk to water resources and supply does vary depending on geographical region,[30] all regions of the United States are exposed to some climate hazards.[31] Climate hazards can pose risks to utility infrastructure systems, operations and maintenance, water quality, and business functions, separate from affecting water supply. Every participant that perceived the issue as one for “others” to contend with did not see climate change as posing a risk to utility business functions. Another institutional limitation is the focus on historical precedent to guide concern for climate hazards. One participant said, “locally here, I don't think a lot of utilities have a lot of plans in place, just because of how fortunate we have been historically.” When discussing whether or not a climate hazard could pose a risk to their system’s reliability, participants relied on recent experience of that hazard.

Table S6. Institutional Limitations to Climate Adaptation Planning Code Frequency

Parent Code	Child Codes	Definition	Total Mentions	% of Participants
Institutional Limitations		Things that limit the utility's capacity for climate planning		
	Don't Want to Limit Growth	Climate adaptation planning perceived as limiting ability to expand system	1	2%
	Haven't Experienced Effects of Climate Change Yet	Utility has not experienced worsening climate change hazards so is not planning for future changes	6	10%
	Focus on Stationarity	Focus on stationarity and using historical data prevents climate planning	1	2%
	No Regulation Requiring Climate Planning	No requirement to complete climate adaptation/resilience plan	2	3%
	No Consensus About Effects	No consistency or certainty in climate change projections	1	2%
	Out of Utility's Control	Believes climate hazards are affecting systems outside utility control so utility can't plan for them	3	5%
	Climate Change is a Political issue	Political factors reduce ability to develop climate adaptation plans	3	5%
	Employee Turnover	Lack of consistent direction within utility reduces ability to develop climate adaptation plans	3	5%
	Problem for Wastewater or Stormwater Utilities	Climate change hazards require adaptation by WW or SW, not DW	12	20%
	Problem in “Other Places"	Climate change hazards pose risks to utilities in other parts of country, this utility is in the clear	15	25%
	Utility Too Small	Utility has limited resources to dedicate to climate planning	1	2%

References

(1) Chester, M.; Markolf, S.; Fraser, A.; Burillo, D.; Bondank, E.; Kim, Y.; Hoehne, C. Infrastructure and Climate Change. *Routledge Handb. Sustain. Resilient Infrastruct.* **2018**, 605–625. https://doi.org/10.4324/9781315142074-31.

(2) Heyn, K.; Winsor, W. *Climate Risks to Water Utility Built Assets and Infrastructure*. Water Utility Climate Alliance. https://www.wucaonline.org/assets/pdf/pubs-asset-infrastructure.pdf (accessed 2021-09-06).

(3) Bondank, E. N.; Chester, M. V.; Ruddell, B. L. Water Distribution System Failure Risks with Increasing Temperatures. *Environ. Sci. Technol.* **2018**, *52* (17), 9605–9614. https://doi.org/10.1021/acs.est.7b01591.

(4) Cole, I. S.; Marney, D. The Science of Pipe Corrosion: A Review of the Literature on the Corrosion of Ferrous Metals in Soils. *Corros. Sci.* **2012**, *56*, 5–16. https://doi.org/10.1016/j.corsci.2011.12.001.

(5) McNeill, L. S.; Edwards, M. The Importance of Temperature in Assessing Iron Pipe Corrosion in Water Distribution Systems. *Environ. Monit. Assess.* **1981**, *77*, 229–242. https://doi.org/https://doi.org/10.1023/a:1016021815596.

(6) Volk, C.; Dundore, E.; Schiermann, J.; Lechevallier, M. Practical Evaluation of Iron Corrosion Control in a Drinking Water Distribution System. *Water Res.* **2000**, *34* (6), 1967–1974. https://doi.org/10.1016/S0043-1354(99)00342-5.

(7) Whittle, A.; Stahmer, M. *Temperature derating of PVC pipes for pressure applications*. Plastics Industry Pipe Association of Australia Limited. https://www.pipa.com.au/wp-content/uploads/2018/09/tn003.pdf (accessed 2021-10-03).

(8) Wols, B. A.; Van Thienen, P. Modelling the Effect of Climate Change Induced Soil Settling on Drinking Water Distribution Pipes. *Comput. Geotech.* **2014**, *55*, 240–247. https://doi.org/10.1016/j.compgeo.2013.09.003.

(9) Wols, B. A.; Van Daal, K.; Van Thienen, P. Effects of Climate Change on Drinking Water Distribution Network Integrity: Predicting Pipe Failure Resulting from Differential Soil Settlement. *Procedia Eng.* **2014**, *70*, 1726–1734. https://doi.org/10.1016/j.proeng.2014.02.190.

(10) Arrighi, C.; Tarani, F.; Vicario, E.; Castelli, F. Flood Impacts on a Water Distribution Network. *Nat. Hazards Earth Syst. Sci.* **2017**, *17* (12), 2109–2123. https://doi.org/10.5194/nhess-17-2109-2017.

(11) Hudak, P. F.; Sadler, B.; Hunter, B. *Analyzing Underground Water- Pipe Breaks in Residual Soils*. Water Engineering and Management. https://img.wwdmag.com/files/base/ebm/wwdmag/document/2022/06/1655271482018-analyzingunderground.pdf (accessed 2021-10-05).

(12) Rotzoll, K.; Fletcher, C. H. Assessment of Groundwater Inundation as a Consequence of Sea-Level Rise. *Nat. Clim. Chang.* **2013**, *3* (5), 477–481. https://doi.org/10.1038/nclimate1725.

(13) Azevedo de Almeida, B.; Mostafavi, A. Resilience of Infrastructure Systems to Sea-Level Rise in Coastal Areas: Impacts, Adaptation Measures, and Implementation Challenges. *Sustain.* **2016**, *8* (11). https://doi.org/10.3390/su8111115.

(14) Pieper, K. J.; Tang, M.; Jones, C. N.; Weiss, S.; Greene, A.; Mohsin, H.; Parks, J.; Edwards, M. A. Impact of Road Salt on Drinking Water Quality and Infrastructure Corrosion in Private Wells. *Environ. Sci. Technol.* **2018**, *52* (24), 14078–14087. https://doi.org/10.1021/acs.est.8b04709.

(15) Sheefa, D. E.; Barkdoll, B. D. Spread of Salt through Municipal Water Distribution Systems. *Environ. Dev. Sustain.* **2022**, No. March. https://doi.org/10.1007/s10668-022-02278-7.
(16) Proctor, C. R.; Lee, J.; Yu, D.; Shah, A. D.; Whelton, A. J. Wildfire Caused Widespread Drinking Water Distribution Network Contamination. *AWWA Water Sci.* **2020**, *2* (4), 1–14. https://doi.org/10.1002/aws2.1183.
(17) Isaacson, K. P.; Proctor, C. R.; Wang, Q. E.; Edwards, E. Y.; Noh, Y.; Shah, A. D.; Whelton, A. J. Drinking Water Contamination from the Thermal Degradation of Plastics: Implications for Wildfire and Structure Fire Response. *Environ. Sci. Water Res. Technol.* **2021**, *7* (2), 274–284. https://doi.org/10.1039/d0ew00836b.
(18) Solomon, G. M.; Hurley, S.; Carpenter, C.; Young, T. M.; English, P.; Reynolds, P. Fire and Water: Assessing Drinking Water Contamination After a Major Wildfire. *ACS ES&T Water* **2021**, *1* (8), 1878–1886. https://doi.org/10.1021/acsestwater.1c00129.
(19) Henry, H. A. L. Climate Change and Soil Freezing Dynamics: Historical Trends and Projected Changes. *Clim. Change* **2008**, *87* (3–4), 421–434. https://doi.org/10.1007/s10584-007-9322-8.
(20) Barton, N. A.; Farewell, T. S.; Hallett, S. H.; Acland, T. F. Improving Pipe Failure Predictions: Factors Effecting Pipe Failure in Drinking Water Networks. *Water Res.* **2019**, *164*, 114926. https://doi.org/10.1016/j.watres.2019.114926.
(21) Bruaset, S.; Sægrov, S. An Analysis of the Potential Impact of Climate Change on the Structural Reliability of Drinking Water Pipes in Cold Climate Regions. *Water (Switzerland)* **2018**, *10* (4). https://doi.org/10.3390/w10040411.
(22) Wasley, E.; Jacobs, K.; Weiss, J.; Preston, N.; Richmond, M. *Mapping Climate Exposure and Climate Information Needs to Water Utility Business Functions. Project 4729*. Water Utility Climate Alliance. https://www.wucaonline.org/assets/pdf/project-4729A-executive-summary.pdf (accessed 2021-09-06).
(23) Water Utility Climate Alliance. *It's Hot and Getting Hotter: Implications of Extreme Heat on Water Utility Staff and Infrastructure , and Ideas for Adapting*. https://www.wucaonline.org/assets/pdf/pubs-implications-of-extreme-heat.pdf (accessed 2021-07-28).
(24) Association of Metropolitan Water Agencies. *Insurance, Bond Ratings and Climate Risk*. https://www.amwa.net/system/files/linked-files/Insurance-BondRatings-ClimateRisk-Paper.pdf (accessed 2021-10-09).
(25) Rubin, S. A Call for Reliability Standards. *J. / Am. Water Work. Assoc.* **2011**, *103* (1), 22–24. https://doi.org/10.1002/j.1551-8833.2011.tb11377.x.
(26) American Water Works Association. *Utility Benchmarking Performance Indicators 2021*. American Water Works Association. https://www.awwa.org/Portals/0/AWWA/ETS/Programs/Benchmarking/2021-Utility-Benchmarking-PIs.pdf?ver=2021-01-05-111236-813 (accessed 2021-09-06).
(27) Berg, S.; Marques, R. Quantitative Studies of Water and Sanitation Utilities: A Benchmarking Literature Survey. *Water Policy* **2011**, *13* (5), 591–606. https://doi.org/10.2166/wp.2011.041.
(28) Bohman, A. Investing in Power System Resilience: A Mixed Methods Approach to Assessing the Tradeoffs of Resilience Strategies., ProQuest Dissertations Publishing, 2022.
(29) Barsugli, J.; Vogel, J.; Kaatz, L.; Smith, J.; Waage, M.; Anderson, C. J. Two Faces of

Uncertainty: Climate Science and Water Utility Planning Methods. *J. Water Resour. Plan. Manag.* **2012**, *138* (5), 389–395. https://doi.org/10.1061/(asce)wr.1943-5452.0000188.
(30) Miller, K. A.; Belton, V. Water Resource Management and Climate Change Adaptation: A Holistic and Multiple Criteria Perspective. *Mitig. Adapt. Strateg. Glob. Chang.* **2014**, *19* (3), 289–308. https://doi.org/10.1007/s11027-013-9537-0.
(31) Jay, A.; Reidmiller, D. R.; Avery, C. W.; Barrie, D.; DeAngelo, B. J.; Dave, A.; Dzaugis, M.; Kolian, M.; Lewis, K. L. M.; Reeves, K.; Winner, D. A. *Chapter 1 : Overview. Impacts, Risks, and Adaptation in the United States: The Fourth National Climate Assessment, Volume II*; Washington, DC, 2018. https://doi.org/10.7930/NCA4.2018.CH1.
